# circFAT1 Promotes Cancer Stemness and Immune Evasion by Promoting STAT3 Activation

**DOI:** 10.1002/advs.202003376

**Published:** 2021-05-02

**Authors:** Lingfei Jia, Yilun Wang, Cun‐Yu Wang

**Affiliations:** ^1^ Jonsson Comprehensive Cancer Center UCLA Los Angeles CA 90095 USA; ^2^ Laboratory of Molecular Signaling Division of Oral Biology and Medicine School of Dentistry UCLA Los Angeles CA 90095 USA; ^3^ Department of Bioengineering Henry Samueli School of Engineering and Applied Science UCLA Los Angeles CA 90095 USA

**Keywords:** cancer stem cells, circFAT1, circRNA, head and neck squamous cell carcinoma, immune evasion, PD1 blockade, STAT3

## Abstract

Cancer stemness and immune evasion are closely associated, and play critical roles in tumor development and resistance to immunotherapy. However, little is known about the underlying molecular mechanisms that coordinate this association. Here, it is reported that elevated circular RNA FAT1 (circFAT1) in squamous cell carcinoma (SCC) unifies and regulates the positive association between cancer stemness and immune evasion by promoting STAT3 activation. circFAT1 knockdown (KD) reduces tumorsphere formation of SCC cells in vitro and tumor growth in vivo. Bioinformatic analysis reveals that circFAT1 KD impairs the cancer stemness signature and activates tumor cell‐intrinsic immunity. Mechanistically, circFAT1 binding to STAT3 in the cytoplasm prevents STAT3 dephosphorylation by SHP1 and promotes STAT3 activation, resulting in inhibition of STAT1‐mediated transcription. Moreover, circFAT1 KD significantly enhances PD1 blockade immunotherapy by promoting CD8^+^ cell infiltration into tumor microenvironment. Taken together, the results demonstrate that circFAT1 is an important regulator of cancer stemness and antitumor immunity.

## Graphical abstract

Cancer stemness and immune evasion are tightly associated with tumor development and immunotherapy resistance. This study shows that circFAT1 controls cancer stemness and immune evasion by regulating STAT3 activation. CircFAT1 binds to STAT3 and prevents STAT3 dephosphorylation by SHP1, resulting in the promotion of STAT3 activation. Knockdown of circFAT1 impairs cancer stemness and enhances PD1 blockade immunotherapy.

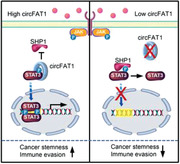

## 1 Introduction

Head and neck squamous cell carcinoma (HNSCC), arising from the oral cavity, oropharynx, larynx, and hypopharynx, is a highly aggressive tumor.^[^ ^1^ ^]^ HNSCC is very invasive and frequently metastasizes to cervical lymph nodes which are enriched with immune cells.^[^ ^2^ ^]^ With the conventional treatment including surgery, radiotherapy, and chemotherapy, the 5‐year survival rate of patients with HNSCC is still relatively low because of tumor relapse, metastasis, and resistance after treatment.^[^ ^2^ ^]^ HNSCC is well‐known for having an immunosuppressive tumor microenvironment with low tumor‐infiltrating lymphocytes.^[^ ^3^ ^]^ Recently, PD1 blockade‐based immunotherapy combined with chemotherapy has been approved as a first‐line of treatment for recurrent or metastatic HNSCC. Unfortunately, the objective response rate remains low in the 20% to 30% ranges, and the median response duration is relatively short, suggesting that HNSCC is resistant to PD1 blockade.^[^ ^4^ ^]^

Cancer stemness and immune evasion have emerged as important features of HNSCC initiation, development, and metastasis. Mounting evidence indicates that cancer stem cells (CSCs) are associated with the initiation, growth, metastasis, relapse, and drug resistance of HNSCC.^[^ ^5^ ^]^ To develop effective strategies in targeting CSCs in HNSCC, we need a better understanding of molecular and epigenetic mechanisms which control CSC properties. A number of studies have focused on the inhibition of regulatory pathways that are critical for the stemness and tumorigenic potentials of CSCs. Interestingly, cancer stemness has been found to be strongly associated tumor cell‐intrinsic immunosuppressive features.^[^ ^6^ ^]^ It is well‐known that immunity plays a critical role in the surveillance against emerging malignant cells from developing into tumors and the inhibition of tumor progression and metastasis.^[^ ^7^ ^]^ CSCs have to develop intrinsic mechanisms to escape from immune surveillance during tumor development and growth. HNSCC frequently metastasizes to cervical lymph nodes, but not other organs, which are enriched with immune cells.^[^ ^2c^ ^]^ Because scattered CSCs mediate metastasis, CSCs should be intrinsically resistant to immune cell killing and then grow in lymph nodes. However, little is known about how cancer stemness and immune evasion are molecularly and epigenetically regulated.

circRNA which forms a closed continuous loop with the 3′ RNA and 5′ RNA joined covalently, is a new type of single‐stranded RNA. Growing evidence suggest that circRNA might regulate various biological processes including signaling transduction, growth, and development.^[^ ^8^ ^]^ While the biological functions of most circRNAs are unclear, there are some studies suggesting that circRNA regulates tumor development and metastasis.^[^ ^9^ ^]^ In HNSCC, a group of circRNAs have been found to be significantly dysregulated.^[^ ^10^ ^]^ However, the functional roles of these circRNAs in HNSCC development and progression have not been explored. Here, we analyzed the expression profile of circRNAs in HNSCC with paired adjacent tissues, and identified that circFAT1 was highly expressed in HNSCC. Intriguingly, we found that circFAT1 controlled cancer stemness and antitumor immunity through STAT3 activation, providing important insights into immune evasion of CSCs in HNSCC.

## 2 Results

### 2.1 Increased circFAT1 in Human HNSCC

**Figure 1:**
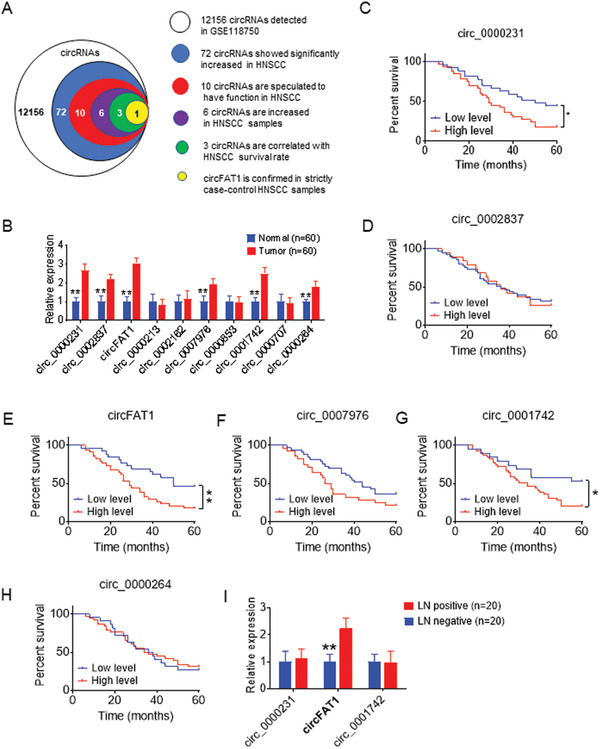
circFAT1 is abnormally expressed in HNSCC and associated with poor prognosis. A) Schematic illustration of identification of circRNAs significantly increased in HNSCC. B) qRT‐PCR analysis of 10 circRNAs in pairs of human HNSCC tissues and adjacent normal tissues. Means ± SD are shown (*n* = 60). **p < 0.01 by paired Student's *t‐*test. C–H) Kaplan–Meier analysis of the overall survival time of HNSCC patients with different circRNAs expression as indicated (*n* = 60). *p < 0.05 and **p < 0.01 by Log‐Rank test. I) qRT‐PCR analysis of circRNAs expression as indicated in a group of HNSCC patients with lymph node metastases (LN positive) compared with a group of patients without lymph node metastases (LN negative). Means ± SD are shown (*n* = 20). **p < 0.01 by paired Student's *t*‐test.

We acquired high‐sequence data of paired HNSCC tumors and adjacent normal tissues from Gene Expression Omnibus (GEO) dataset (GEO number: GSE118750),^[^ ^10c^ ^]^ and reperformed bioinformatic analysis of differently expressed circRNAs (**Figure** **1**A) with more stringent conditions. Fold change >3.0 and *p* < 0.02 were used as cutoffs for analyzing 4573 circRNAs in GSE118750, and 72 circRNAs were found to be highly expressed in HNSCC compared with adjacent normal tissues (Figure S1, Supporting Information). Among them, 7 circRNAs (circ_0001461 named as circFAT1, circ_0000231, circ_0000213, circ_0000853, circ_0001742, circ_0000707, and circ_0000264) have been annotated as “cancer‐related” in Circ2Traits database,^[^ ^11^ ^]^ and 3 circRNAs (circ_0002837, circ_0002162, and circ_0007976) satisfied the condition that their host gene (mRNA) participated in classic tumor signal pathway. The above 10 circRNAs expression was further tested in 60 pairs of HNSCC tumor tissues and adjacent normal tissues using quantitative reverse transcriptase‐polymerase chain reaction (qRT‐PCR). The results showed that 6 circRNAs (circFAT1, circ_0000231, circ_0001742, circ_0000264, circ_0002837, circ_0007976) were significantly increased in tumor tissues compared with corresponding normal tissues (Figure 1B). The Kaplan–Meier survival analysis showed significantly shorter overall survival times of HNSCC patients with elevated expression levels of circ_0000231, circFAT1, and circ_0001742 compared with patients with low expression levels (Figure 1C–H).

HNSCC frequently metastasize to cervical lymph nodes, and the poor prognosis of the HNSCC patients is closely associated with lymph node metastasis.^[^ ^3^ ^]^ Since patient clinical characteristics, such as age, gender, pathologic differentiation, and cancer stage, may influence HNSCC lymph node metastasis and prognosis,^[^ ^12^ ^]^ we designed strict case‐control studies to exclude the influence of these clinical factors. We collected tumor tissues from 40 patients with HNSCC matched for age, gender, pathologic differentiation, and cancer stage which included a group of 20 HNSCC patients without lymph node metastases who had survived over 5 years after operation (LN negative group) and a group of 20 HNSCC patients with lymph node metastases most of whom survived less than 4 years after operation (LN positive group) (Table S1, Supporting Information). The expression of circ_0000231, circFAT1, and circ_0001742 were further analyzed and compared in both groups. The results showed that circFAT1 was significantly increased in the LN positive group compared with the LN negative group (Figure 1I). We therefore chose circFAT1 for further investigation.

### 2.2 Promoting HNSCC Invasive Growth by circFAT1

**Figure 2:**
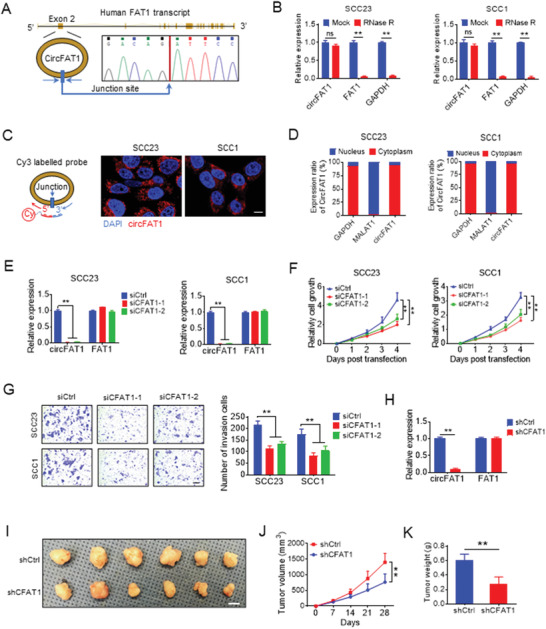
circFAT1 KD inhibits HNSCC growth and invasion. A) Schematic illustration showing that the Human FAT1 exon 2 circularization forms circFAT1. The presence of circFAT1 was validated by qRT‐PCR, followed by Sanger sequencing. Blue arrow represents “head‐to‐tail” circFAT1 splicing site. B) qRT‐PCR analysis of the expression *circFAT1*, *FAT1*, and *GAPDH* in SCC23 and SCC1 cells treated with or without RNase R. Means ± SD are shown. ns, not significant. **p < 0.01 by unpaired Student's *t*‐test. C) Identification of circFAT1 cytoplasmic distribution by FISH in SCC23 and SCC1 cells. The left panel show the circFAT1 probe was designed to target junction site and labeled with Cy3. Nuclei were stained with DAPI. Scale bar: 10 µm. D) circFAT1 cytoplasmic distribution by qRT‐PCR analysis in SCC23 and SCC1 cells. *GAPDH* and *MALAT1* were used as the cytoplasmic and nuclear markers, respectively. E) qRT‐PCR analysis of *circFAT1* and *FAT1* expression in SCC23 and SCC1 cells treated with siRNA control (siCtrl) or circFAT1 (siCFAT1‐1 and siCFAT1‐2) 24 h after transfection. Means ± SD are shown. **p < 0.01 by unpaired Student's *t*‐test. F) Effect of circFAT1 KD on proliferation in SCC23 and SCC1 cells. Means ± SD are shown. **p < 0.01 by unpaired Student's *t*‐test. G) Effect of circFAT1 KD on invasion in SCC23 and SCC1 cells in 24 h. Scale bar: 50 µm. Means ± SD are shown. **p < 0.01 by unpaired Student's *t*‐test. H) Expression levels of *circFAT1* and *FAT1* in SCC23 cells transduced with shRNA circFAT1 (shCFAT1). Means ± SD are shown. **p < 0.01 by unpaired Student's *t*‐test. I) The image of SCC23 xenografted tumors in nude mice after circFAT1 KD. Scale bar: 1 cm. J) circFAT1 KD inhibited SCC23 tumor growth in mice. Means ± SD are shown (*n* = 6). **p < 0.01 by unpaired Student's *t*‐test. K) SCC23 tumor weight after circFAT1 KD. Means ± SD are shown (*n* = 6). **p < 0.01 by unpaired Student's *t*‐test.

We confirmed head‐to‐tail splicing in the circFAT1 qRT‐PCR product, along with the circFAT1 size by Sanger sequencing (**Figure** **2**A). Using cDNA and genomic DNA from SCC23 and SCC1 cells as templates, circFAT1 was amplified by divergent primers in cDNA, but not in genomic DNA (Figure S2A, Supporting Information). PCR analysis showed that the poly A‐tailed mRNAs *FAT1* and *GAPDH* could be reversed to cDNA by using random or oligo (dT) primer, and the non‐poly A‐tailed RNA circFAT1 could not be reversed to cDNA by using oligo (dT) primer (Figure S2B, Supporting Information). circFAT1 was resistant to RNase R treatment, while the linear RNAs *FAT1* and *GAPDH* were substantially digested with RNase R (Figure 2B; Figure S2C, Supporting Information). Using a Cy3‐labeled circFAT1‐specific probe to target the junction region, RNA fluorescence in situ hybridization (FISH) assays demonstrated that circFAT1 was predominately localized in the cytoplasm (Figure 2C). The nuclear/cytoplasm fractionation further confirmed that circFAT1 was mainly expressed in the cytoplasm (Figure 2D). As a control, *GAPDH* was found in the cytoplasm, and metastasis associated lung adenocarcinoma transcript 1 (*MALAT1*), which is known to be highly expressed in the nucleus, was detected in the nucleus.

To explore the functional role of circFAT1 in HNSCC, we used two sets of small‐interfering RNAs (siRNAs) siCFAT1‐1 and siCFAT1‐2 specifically targeting the junction site of circFAT1. circFAT1 KD by siCFAT1‐1 and ciCFAT1‐2 significantly reduced the expression of circFAT1, but not *FAT1* in SCC23 and SCC1 cells (Figure 2E; Figure S2D, Supporting Information). circFAT1 KD partially reduced the proliferation of SCC23 and SCC1 cells (Figure 2F). Matrigel invasion assays revealed that circFAT1 KD also significantly inhibited the invasion ability of SCC23 and SCC1 cells (Figure 2G). To further examine whether circFAT1 promoted tumor growth in vivo, we knocked down circFAT1, but not FAT1 in SCC23 via lentivirus‐based short‐hairpin RNA for circFAT1 (shCFAT1) (Figure 2H; Figure S2E–G, Supporting Information). circFAT1 KD by shCFAT1 significantly reduced the volume and weight of SCC23 cell‐derived tumors grown in nude mice compared with SCC23 cells transfected with shRNA control (shCtrl) (Figure 2I–K).

### 2.3 Controlling Cancer Stemness Signature by circFAT1

**Figure 3:**
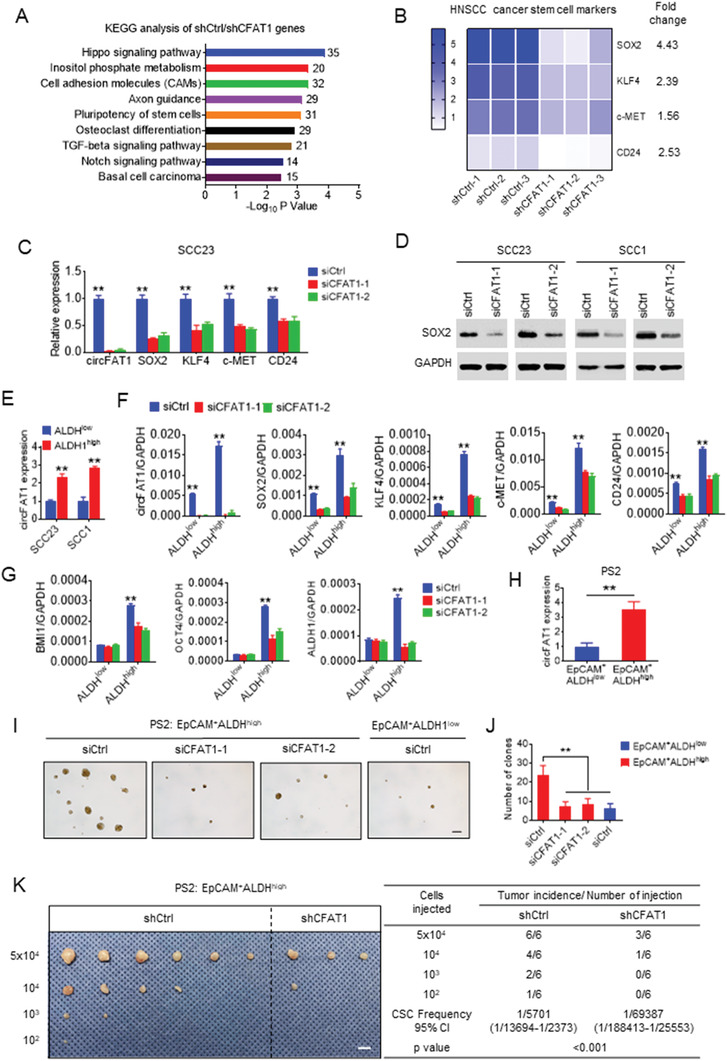
circFAT1 controls the tumorigenic potential of CSCs in HNSCC. A) Histogram of the top 9‐enriched KEGG pathways of upregulated genes (shCtrl/shCFAT1, fold change > 1.5) in SCC23 cells. *Y*‐axis, KEGG pathway categories; *X*‐axis, statistical significance of the enrichment. Numbers of genes belonging to each KEGG category are listed in right. B) Heatmap from RNA‐sequencing data showing the expression of HNSCC stem cells markers as indicated in SCC23 cells transfected with shCtrl or shCFAT1. Fold change of shCtrl/shCFAT1 was listed in the right. C) qRT‐PCR analysis of the expression of HNSCC cancer stem cells markers in ALDH^high^ cells upon circFAT1 KD. Means ± SD are shown. **p < 0.01 by unpaired Student's *t*‐test. D) Western blot analysis of SOX2 in SCC23 and SCC1 cells with circFAT1 KD. E) qRT‐PCR analysis of the circFAT1 expression in ALDH^high^ and ALDH^low^ cells. Means ± SD are shown. **p < 0.01 by unpaired Student's *t*‐test. F) qRT‐PCR analysis *circFAT1, SOX2, KLF4, c‐MET*, and *CD24* expression in ALDH^low^ and ALDH^high^ SCC23 cells with circFAT1 KD. The relative expression level of *circFAT1, SOX2, KLF4, c‐MET*, and *CD24* were normalized with *GAPDH* by the 2^−ΔCt^ method. Means ± SD are shown. **p < 0.01 by unpaired Student's *t*‐test. G) qRT‐PCR analysis of *BMI1, OCT4*, and *ALDH1* expression in ALDH^low^ and ALDH^high^ SCC23 cells with circFAT1 knockdown. The relative expression level of *BMI1, OCT4*, and *ALDH1* were normalized with *GAPDH* by the 2^−ΔCt^ method. Means ± SD are shown. **p < 0.01 by unpaired Student's *t*‐test. H) qRT‐PCR analysis of the expression of *circFAT1* in EpCAM^+^ALDH^high^ cells isolated from HNSCC patient case #2 derived xenograft (PS2). Means ± SD are shown. **p < 0.01 by unpaired Student's *t*‐test. I) Representative image of tumorspheres formation of EpCAM^+^ALDH^high^ and EpCAM^+^ALDH^low^ tumor cells from PS2 transfected with siRNAs as indicated. Scale bar: 100 µm. J) Quantification of tumorspheres from EpCAM^+^ALDH^high^ and EpCAM^+^ALDH^low^ tumor cells from PS2 transfected with siRNAs as indicated. Means ± SD are shown. **p < 0.01 by unpaired Student's *t*‐test. K) In vivo limiting dilution analysis of EpCAM^+^ALDH^high^ tumor cells from PS2 transfected with shCtrl and shCFAT1 (*n* = 6). The frequency of allograft formation at each cell dose injected was shown. The data were analyzed using ELDA software. Scale bar: 1 cm.

To explore the underlying mechanisms by which circFAT1 KD inhibited HNSCC growth, we performed the whole transcriptome analysis of SCC23 cells upon circFAT1 KD using RNA sequencing. Kyoto encyclopedia of genes and genomes (KEGG) analysis revealed that circFAT1 KD affected the expression of genes enriched in the “pluripotency of stem cells” gene term (**Figure** **3**A) in addition to the signaling pathways associated with cell growth, migration and invasion. The expression profiles of *SOX2, KLF4, MET*, and *CD24*, which were associated with HNSCC CSCs signatures,^[^ ^5^, ^13^ ^]^ were exhibited using Heatmap (Figure 3B). qRT‐PCR confirmed that circFAT1 KD reduced the expression of *SOX2, KLF4, MET*, and *CD24* in SCC23 cells (Figure 3C). As one of the most significantly downregulated genes, SOX2 inhibited by circFAT1 KD in SCC23 and SCC1 cells was also confirmed by Western blot (Figure 3D). We further examined whether SOX2 expression was associated with circFAT1 in human HNSCC by quantitative reverse transcriptase‐polymerase chain reaction (qRT‐PCR), and Pearson's rank correlation coefficient analysis also revealed a positive correlation between *circFAT1* and *SOX2* mRNA expression using 60 of frozen HNSCC tissue samples (Figure S3A, Supporting Information). Previously, studies have shown that CSCs from human primary HNSCC tissues or SCC cell lines have elevated aldehyde dehydrogenase (ALDH) activities.^[^ ^14^ ^]^ Using an ALDEFLUOR kit, we sorted ALDH^low^ nonstem tumor cells and ALDH^high^ CSC‐like cells with the specific ALDH inhibitor diethylaminobenzaldehyde (DEAB) as a control (Figure S3B, Supporting Information), and found that the circFAT1 expression was significantly higher in ALDH^high^ CSCs than in ALDH^low^ tumor cells (Figure 3E). qRT‐PCR showed that circFAT1 KD significantly inhibited the expression of the cancer stemness‐related genes, including *SOX2, KLF4, MET, BMI1, CD24, OCT4*, and *ALDH1*, in ALDH^high^ SCC23 cells (Figure 3F,G). Although the expression of *SOX2, KLF4, MET*, and *CD24* were relatively low in ALDH^low^ SCC23 cells, circFAT1 KD also reduced their expression in ALDH^low^ SCC23 cells (Figure 3F). circFAT1 KD did not significantly affect the expression of *BMI1, OCT4*, and *ALDH1* in ALDH^low^ SCC23 cells probably due to the fact that the basal levels of these genes were very low in ALDH^low^ SCC23 cells (Figure 3G). The tumorsphere formation assay is a simple method to measure the self‐renewal of CSCs in vitro. circFAT1 KD also significantly inhibited the tumorsphere formation ability of ALDH^high^ SCC23 cells (Figure S3C,D, Supporting Information). Moreover, in vivo limiting dilution tumorigenicity assays showed that circFAT1 KD inhibited tumorigenic potentials of ALDH^high^ SCC23 cells in nude mice (Figure S3E, Supporting Information). To further confirm whether circFAT1 controlled the tumorigenic potential of CSCs, we isolated CSCs from HNSCC patient‐derived xenograft (PDX) using EpCAM^+^ALDH^high^ markers as described previously.^[^ ^14^ ^]^ Consistently, qRT‐PCR found that circFAT1 was higher in EpCAM^+^ALDH^high^ CSCs than in EpCAM^+^ALDH^low^ nonstem tumor cells (Figure 3H). circFAT1 KD also inhibited the tumorspheres formation of EpCAM^+^ALDH^high^ CSCs in vitro (Figure 3I,J). In vivo limiting dilution tumorigenicity assays showed that circFAT1 KD also inhibited tumorigenic potentials of EpCAM^+^ALDH^high^ CSCs in nude mice (Figure 3K).

### 2.4 Enhancing STAT3 Activation by circFAT1

**Figure 4:**
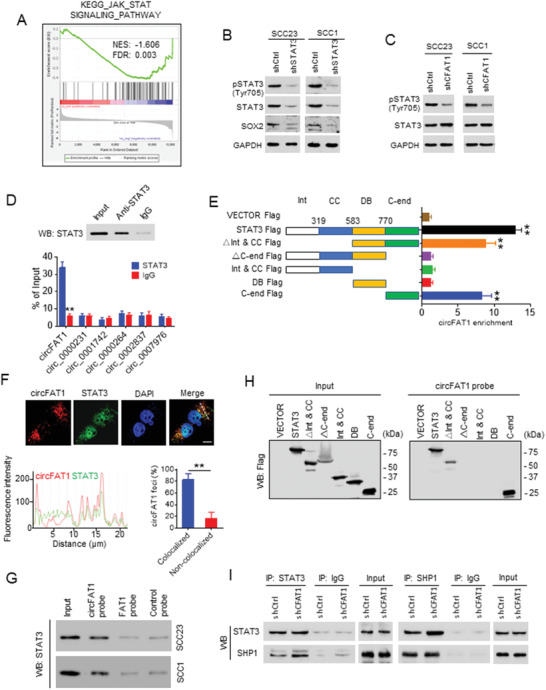
circFAT1 interacts with STAT3 and prevents STAT3 binding to SHP1. A) GSEA analysis showed that the JAK‐STAT signaling pathway related genes were significantly enriched in SCC23 with circFAT1 KD. The colored panel at the bottom indicates the level of differential expression from the most upregulated (red) to most downregulated (blue). B) Western blot analysis of the pSTAT3, STAT3, and SOX2 in SCC23 and SCC1 cells with STAT3 KD. C) Western blot analysis of pSTAT3 and STAT3 in SCC23 and SCC1 cells with circFAT1 KD. D) qRT‐PCR detection of circFAT1, circ_0000231, circ_0001742, circ_0000264, circ_0002837, and circ_0007976 pulled down by anti‐STAT3 compared with IgG control. Top panel: anti‐STAT3 antibodies were confirmed by Western blot (WB). Means ± SD are shown. **p < 0.01 by unpaired Student's *t*‐test. E) qRT‐PCR detection of *circFAT1* retrieved by Flag‐tagged full‐length or domain truncated STAT3 using anti‐Flag. Int, protein interaction domain; CC, coiled‐coil domain; DB, DNA binding domain; C‐end, C‐terminus. F) Colocalization of circFAT1 and STAT3 in SCC23 cells by a confocal microscopy. Scale bar: 10 µm (upper panel). The fluorescent intensity was quantified along with the indicated white dashed line in cells. The foci of circFAT1 and STAT3 were counted from a total of 50 cells (lower panel). circFAT1 foci were divided into two groups based on its colocalization or non‐colocalization with STAT3. Means ± SD are shown. *n* = 50, **p < 0.01 by paired Student's *t*‐test. G) RNA pull‐down assay detected the interactions between circFAT1 and STAT3 by Western blot (WB) in SCC23 and SCC1 cells. H) Western blot (WB) analysis showing that circFAT1 retrieved Flag‐tagged full‐length or domain truncated STAT3 using RNA pull‐down assays. I) Western blot (WB) analysis of indicated proteins in STAT3‐immunoprecipitated complexes (left panel) or in SHP1‐immunoprecipitated complexes (right panel) from lysates of SCC23 with circFAT1 KD.

circRNA–protein interactions have been reported to influence protein expression and function.^[^ ^15^ ^]^ circFAT1 was located primarily in the cytoplasm, suggesting circFAT1 might exert its biological function in the cytoplasm. Since SOX2, KLF4, and BMI1 are transcription factors which are mainly located in the nucleus of cancer cells, it was unlikely that circFAT1 regulated their expression directly. Thus, we searched for the potential upstream signaling pathways, which might regulate the expression of stemness genes. Several oncogenic signaling pathways, including JAK/STAT, PI3K/AKT, WNT, P53, and NF‐*κ*B, have been found to regulate cancer stemness.^[^ ^16^ ^]^ Gene set enrichment analysis (GSEA) showed that the JAK/STAT signaling pathway‐related genes, but not PI3K/AKT, WNT, P53, and NF‐kB, were affected in SCC23 cells with circFAT1 KD (**Figure** **4**A; Figure S4A, Supporting Information). The transcription factor STAT3 is the primary signaling molecule in the JAK/STAT3 pathway, and the activation of STAT3 is associated with CSCs in HNSCC,^[^ ^16^, ^17^ ^]^ Western blot showed that the knockdown of STAT3 by shRNA drastically reduced the level of STAT3 and phospho‐STAT3 (Tyr705) (pSTAT3) and inhibited the expression of SOX2 in SCC23 and SCC1 cells (Figure 4B). Interestingly, we found that circFAT1 KD reduced the levels of pSTAT3 and the nucleus translocation of STAT3, but not the whole levels of STAT3, suggesting that circFAT1 might control STAT3 phosphorylation and activation (Figure 4C; Figure S4B, Supporting Information). In response to external stimuli, STAT3 is phosphorylated at tyrosine 705 by receptor‐associated Janus kinase, and subsequently pSTAT3 is translocated to the nucleus where they activate the target gene transcription. To determine the precise mechanism underlying the regulation of STAT3 phosphorylation by circFAT1, we performed RNA immunoprecipitation (RIP) assays and identified STAT3 as a circFAT1 associated protein (Figure 4D). To analyze the specificity of the interaction, we also examined the levels of circ_0000231, circ_0001742, circ_0000264, circ_0002837, and circ_0007976, which proved highly expressed in HNSCC as described above. The results showed that anti‐STAT3 antibodies did not pull down these circRNAs, suggesting specific interaction between circFAT1 and STAT3 (Figure 4D). Next, RIP assay with full‐length or truncated STAT3 demonstrated that the C terminus of STAT3 interacted with circFAT1 (Figure 4E). Confocal microscopy for circFAT1 FISH and STAT3 immunostaining showed their colocalization in the cytoplasm of SCC23 (Figure 4F). The interaction was further confirmed through RNA pull‐down assay with biotin labeled circFAT1 probe in SCC23 and SCC1 cells (Figure 4G). We also found that STAT3 was rarely pulled down by circFAT1 probes in SCC23 treated with shCFAT1, suggesting the interaction specificity of the circFAT1 probe with STAT3 (Figure S4C, Supporting Information). RNA pull‐down assays with full‐length or truncated STAT3 also demonstrated that circFAT1 interacted with the C terminus of STAT3 (Figure 4H). The phosphorylation of STAT3 is negatively regulated by SHP1, a protein tyrosine phosphatase.^[^ ^18^ ^]^ We further investigated whether STAT3‐binding circFAT1 might interfere with the interaction between SHP1 and STAT3. Co‐immunoprecipitation (IP) experiments showed that STAT3 pulled down increased levels of SHP1 in SCC23 cells with circFAT1 KD compared with control shRNA KD or vice versa (Figure 4I). Taken together, our results suggest that circFAT1 helps to maintain the levels of STAT3 phosphorylation by preventing SHP1‐mediated dephosphorylation of STAT3.

**Figure 5:**
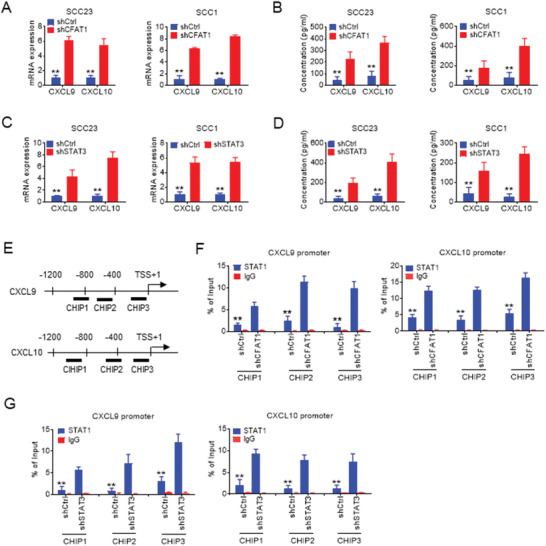
circFAT1 knock down induces the Type 1 IFN chemokines. A) qRT‐PCR analysis of *CXCL9* and *CXCL10* in SCC23 and SCC1 cells with circFAT1 KD. Means ± SD are shown. **p < 0.01 by unpaired Student's *t*‐test. B) ELISA analysis of the protein levels of CXCL9 and CXCL10 secreted by SCC23 and SCC1 cells with circFAT1 KD. Means ± SD are shown. **p < 0.01 by unpaired Student's *t*‐test. C) qRT‐PCR analysis of *CXCL9* and *CXCL10* in SCC23 and SCC1 cells with STAT3 KD. Means ± SD are shown. **p < 0.01 by unpaired Student's *t*‐test. D) ELISA analysis of the protein levels of CXCL9 and CXCL10 secreted by SCC23 and SCC1 cells with STAT3 KD. Means ± SD are shown. **p < 0.01 by unpaired Student's *t*‐test. E) The primer locations on the *CXCL9* and *CXCL10* promoters for ChIP‐qPCR. TSS, Transcription start site. F) ChIP analysis of STAT1 occupancy at the *CXCL9* and *CXCL10* promoters in SCC23 cells with circFAT1 KD. Means ± SD are shown. **p < 0.01 by unpaired Student's *t*‐test. G) ChIP analysis of STAT1 occupancy at the *CXCL9* and *CXCL10* promoters in SCC23 cells with STAT3 KD. Means ± SD are shown. **p < 0.01 by unpaired Student's *t*‐test.

It has been reported that the type I interferon (IFN)‐induced chemokines, CXCL9 and CXCL10, are frequently associated with recruitment of CD8^+^ T lymphocytes to tumor sites.^[^ ^19^ ^]^ It has been shown that pSTAT3 interacts with STAT1 to inhibit type I IFN response.^[^ ^20^ ^]^ To test this possibility, we examined whether the expression of CXCL9 and CXCL10 were upregulated upon circFAT1 KD. qRT‐PCR revealed that circFAT1 KD significantly increased the expression of *CXCL9* and *CXCL10* in SCC23 and SCC1 cells (**Figure** **5**A). The enzyme‐linked immunosorbent assay (ELISA) also confirmed that circFAT1 KD increased the protein levels of CXCL9 and CXCL10 that were secreted by SCC23 and SCC1 cells (Figure 5B). Consistently, STAT3 KD also showed similar results (Figure 5C,D). STAT3 can form a heterodimer with STAT1 and prevent it from forming functional homodimers, thereby inhibiting STAT1‐mediated transcription.^[^ ^21^ ^]^ Next, we investigated whether circFAT1 KD‐induced expression of *CXCL9* and *CXCL10* was due to changes in STAT1 occupancies in their promoters. Western blot showed that neither circFAT1 KD nor STAT3 KD could affect the expression of STAT1 and pSTAT1 (Figure S5A, Supporting Information). Chromatin immunoprecipitation‐quantitative PCR (ChIP‐qPCR) was performed to examine STAT1 occupancies on three potential sites of the *CXCL9* and *CXCL10* promoters (Figure 5E). ChIP‐qPCR revealed that circFAT1 KD significantly increased STAT1 occupancies on the promoters of *CXCL9* and *CXCL10* (Figure 5F). Consistently, STAT3 KD also increased STAT1 occupancies on the promoters of *CXCL9* and *CXCL10* (Figure 5G). PD‐L1 is known to be regulated by IFN signaling, and PD‐L1 induction suppresses the functional activity of tumor infiltrating CD8^+^ T cells.^[^ ^22^ ^]^ We also found that PD‐L1 expression was upregulated in SCC23 and SCC1 with circFAT1 KD (Figure S5B, Supporting Information). Taken together, these findings illustrate that abnormal expression of circFAT1 controls cancer stemness and immune evasion by promoting STAT3 activation.

### 2.5 circFat1 KD Impairs CSCs and Enhances PD1 Blockade Immunotherapy

**Figure 6:**
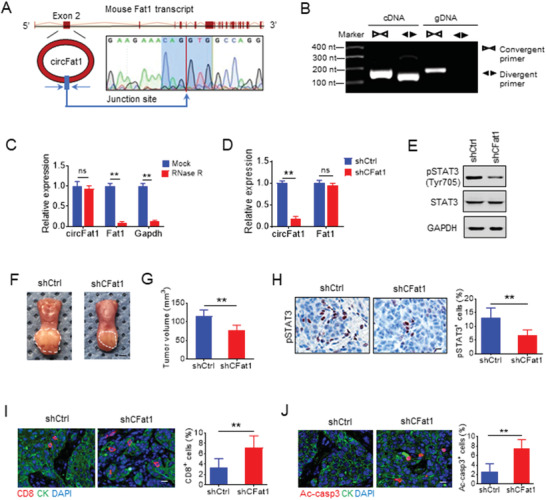
circFat1 knockdown stimulates tumor cell‐intrinsic immune response. A) Schematic illustration showed that the mouse Fat1 exon 2 circularization formed circFat1. The presence of circFat1 was validated by qRT‐PCR, followed by Sanger sequencing. Blue arrow represents “head‐to‐tail” circFat1 splicing sites. B) RT‐PCR products with divergent primers showing circularization of mouse circFat1 in cDNA, but not in genomic DNA. cDNA, complementary DNA. gDNA, genomic DNA. C) qRT‐PCR analysis of circFat1, *Fat1*, and *GAPDH* expression in MOC1 cells treated with or without RNase R. Means ± SD are shown. ns, not significant. **p < 0.01 by unpaired Student's *t*‐test. D) qRT‐PCR analysis of *circFat1* and *Fat1* in MOC1 cells with shCtrl and shCFat1. Means ± SD are shown. ns, not significant. **p < 0.01 by unpaired Student's *t*‐test. E) Western blot analysis of pSTAT3 in MOC1 cells with circFat1 KD. F) Representative images of MOC1‐derived tumors grown in the tongue of C57BL/6J mice upon circFAT1 KD. White dashed lines demark visible lesion areas. Scale bar: 2 mm. G) Quantification of tumor volume from mice with circFAT1 KD. Means ± SD are shown (*n* = 8). **p < 0.01 by unpaired Student's *t*‐test. H) Immunostaining and quantification of pSTAT3^+^ cells in tumors with circFAT1 KD. Scale bar: 10 µm. Means ± SD are shown (*n* = 8). **p < 0.01 by unpaired Student's *t*‐test. I) Immunofluorescent staining and quantification of CD8^+^ T cells in tumor after circFAT1 KD. CK, Pan cytokeratin. Means ± SD are shown (*n* = 8). **p < 0.01 by unpaired Student's *t*‐test. J) Immunofluorescent staining and quantification of active caspase3 (ac‐casp3) in tumors with treatment as indicated. Values are mean ± SD (*n* = 8). **p < 0.01 by unpaired Student's *t*‐test.

The sequences of human circFAT1 and mouse circRNA Fat1 (circFat1) are highly conserved (Table S2, Supporting Information), suggesting conservation in their physiological functions. We confirmed head‐to‐tail splicing in the mouse circFat1 qRT‐PCR product from the murine HNSCC cell line MOC1,^[^ ^23^ ^]^ along with the circFat1 size by Sanger sequencing (**Figure** **6**A). Using cDNA and genomic DNA from the MOC1 cells as templates, circFAT1 was only amplified by divergent primers in cDNA, but not in genomic DNA (Figure 6B). PCR analysis showed that the poly A‐tailed mRNAs *Fat1* and *GAPDH* could be reversed to cDNA by using a random or oligo (dT) primer, while non‐poly A‐tailed RNA circFat1 could not be reversed to cDNA by using an oligo (dT) primer (Figure S6A, Supporting Information). Mouse circFat1 was also resistant to RNase R, while mouse linear mRNAs *Fat1* and *GAPDH* degraded after RNase R treatment (Figure 6C; Figure S6B, Supporting Information). shRNA targeting the junction site of mouse circFat1 (shCFat1) reduced circFat1 expression, but not *Fat1* mRNA (Figure 6D; Figure S6C, Supporting Information). Consistently, the protein levels of pSTAT3, but not STAT3, was also reduced in MOC1 cells expressing shCFat1 (MOC1/shCFat1), but not in MOC1 cells expressing shCtrl (MOC1/shCtrl) (Figure 6E). To investigate the functional role of circFat1 in CSCs and immunogenicity in vivo, we orthotopically transplanted both MOC1/shCtrl and MOC1/shCFat1 cells into the tongue of immunocompetent C57BL/6J mice. The results showed that tumor volume of MOC1/shCFat1 cells was reduced compared with MOC1/shCtrl cells after 4 weeks of orthotopical transplantation (Figure 6F,G). Immunostaining found that circFat1 KD significantly reduced pSTAT3 in mouse HNSCC (Figure 6H). Notably, we found that circFat1 KD significantly increased CD8^+^ T cells infiltration into tumors (Figure 6I), and induced more apoptosis in tumor cells (Figure 6J).

**Figure 7:**
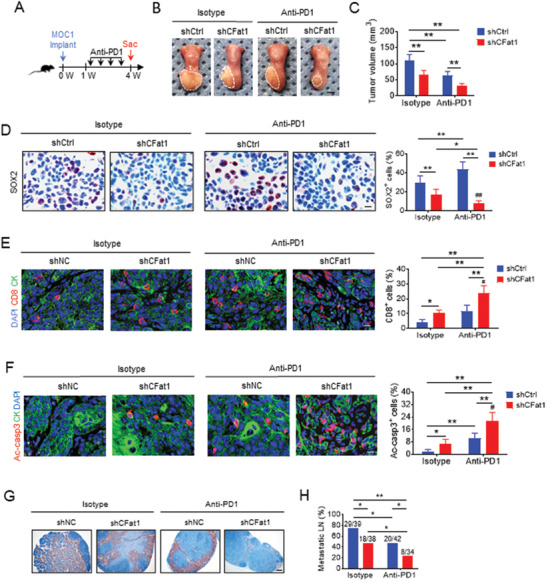
circFat1 KD potentiates PD‐1 blockade immunotherapy in HNSCC. A) Schematic diagram shows the time point of anti‐PD1 treatment and sacrificed (Sac) of mice implanted with MOC1 cells. B) Representative images of MOC1 cell‐derived tumors in C57BL/6J mice after anti‐PD1 treatment. White dashed lines demark visible lesion areas. Scale bar: 2 mm. C) Quantification of tumor volume from mice with treatment as indicated. Means ± SD are shown (*n* = 8). **p < 0.01 by two‐way ANOVA. D) Immunostaining and quantification of SOX2^+^ cells in tumors from mice with treatment as indicated. Scale bar: 10 µm. Means ± SD are shown (*n* = 8). **p < 0.01 by two‐way ANOVA. ##*p* < 0.01 treatment x genotype interaction. E) Immunofluorescent staining and quantification of CD8^+^ T cells in HNSCC upon anti‐PD1 treatment. CK, Pan cytokeratin. Means ± SD are shown (*n* = 8). *p < 0.05 and **p < 0.01 by two‐way ANOVA. #*p* < 0.05 treatment x genotype interaction. F) Immunofluorescent staining and quantification of active caspase3 (ac‐casp3) in HNSCC after anti‐PD1 treatment. Means ± SD are shown (*n* = 8). *p < 0.05 and **p < 0.01 by two‐way ANOVA. #*p* < 0.05 treatment x genotype interaction. G) Immunostaining of metastatic cells in cervical lymph nodes from mice using antipan cytokeratin. Scale bar: 200 µm. H) Percentage of metastatic lymph nodes. Number of metastatic lymph nodes and total lymph nodes in each group is indicated in the figure. Means ± SD are shown (*n* = 8). *p < 0.05 and **p < 0.01 by Chi‐square test.

It was also reported that SOX2 potentiates an immunosuppressive microenvironment and promotes HNSCC growth in vivo by inhibiting type 1 IFN signaling.^[^ ^24^ ^]^ Moreover, SOX2^+^ tumor cells have been identified as CSCs and progenitor cells in SCC. Since circFat1 KD attenuated STAT3 activation and SOX2 expression, we further examined whether circFat1 KD could enhance PD1 blockade‐mediated immunotherapy of HNSCC by activating tumor cell intrinsic type 1 IFN signaling. Both MOC1/shCtrl and MOC1/shCFat1 cells were inoculated into mouse tongue. Whereas anti‐PD1 treatment moderately reduced the tumor growth of MOC1/shCtrl cells, circFAT1 KD further significantly enhanced the inhibition of tumor growth mediated by anti‐PD1 treatment (**Figure** **7**A–C). Interestingly, immunostaining found that, while circFat1 reduced SOX2^+^ tumor cells, SOX2^+^ tumor cells were enriched after anti‐PD1 treatment, indicating that SOX2^+^ tumor cells were intrinsically resistant to PD1 blockade. By contrast, circFat1 KD dramatically potentiated the elimination of SOX2^+^ cells upon anti‐PD1 treatment (Figure 7D). Immunostaining revealed that circFat1 KD, but not anti‐PD1 treatment, increased the protein levels of CXCL10 in tumor cells of HNSCC (Figure S7A, Supporting Information). Consistently, we found that circFat1 KD plus anti‐PD1 significantly recruited more CD8^+^ T cells into the tumor microenvironment compared with anti‐PD1 or circFat1 KD alone (Figure 7E). Cytotoxic CD8^+^ T cells generated Granzyme B (GzmB) to kill tumor cells. Immunostaining with anti‐GzmB demonstrated that circFat1 KD plus anti‐PD1 also recruited more GzmB^+^ CD8^+^ T cells into tumor tissues compared with anti‐PD1 or circFat1 KD alone (Figure S7B,C, Supporting Information). Consistently, circFat1 KD plus anti‐PD1 significantly induced apoptosis in HNSCC compared with circFat1 KD or anti‐PD1 treatment alone, as determined by immunostaining of active caspase‐3 (Figure 7F). Previously, it has been shown that MOC1 cells could not metastasize to cervical lymph nodes by H&E staining.^[^ ^23^ ^]^ However, we did find that MOC1 cells could metastasize to cervical lymph nodes by using immunostaining with antipan cytokeratin that was able to clearly decipher epithelial metastatic tumor cells from lymphocytes in cervical lymph nodes. Immunostaining revealed that circFat1 plus anti‐PD1 treatment exhibited the superior inhibitory effect on lymph node metastasis as compared to anti‐PD1 or circFat1 KD alone (Figure 7G,H).

## 3 Discussion

Growing evidence demonstrates that cancer stemness and immune evasion play a critical role in tumor development, progression, and metastasis.^[^ ^6^ ^]^ circRNAs are now widely recognized as a novel subset of endogenous RNAs that regulate target genes by affecting the functions of miRNAs or forming complexes with target proteins.^[^ ^25^ ^]^ In this study, we identified that circFAT1 was highly expressed in HNSCC which was associated with HNSCC metastasis and poor prognosis. circFAT1 coordinately controlled cancer stemness and immune evasion through promoting STAT3 activation. Mechanistically, circFAT1 bound to STAT3 and promoted STAT3 activation by preventing SHP1‐mediated dephosphorylation of pSTAT3. The knockdown of circFAT1 inhibited tumorigenesis by impairing cancer stemness and reversed tumor immunosuppressive microenvironment. More importantly, we found that circFAT1 KD also helped to overcome immunotherapy resistance of HNSCC to PD1 blockade. These results reveal an important link between circRNA regulation of cancer stemness and molding of an immunosuppressive microenvironment, and it identifies circFAT1 as an important therapeutic target in HNSCC.

Immune surveillance is critical for preventing tumor development and progression. Moreover, HNSCC tumor cells, most likely CSCs, frequently metastasize to and grow in cervical lymph nodes which are enriched with lymphocytes. CSCs probably create some unique mechanisms to evade immune surveillance. Therefore, promoting CSC stemness and immune evasion plays a critical role in HNSCC development and progression. Our studies provided a molecular insight into the stemness and immune evasion of HNSCC. It has been reported that SOX2 potentiates an immunosuppressive microenvironment and promotes HNSCC growth in vivo in a type 1 IFN signaling‐dependent fashion.^[^ ^24^ ^]^ Interestingly, SOX2^+^ tumor cells have been identified as CSCs in the skin SCC.^[^ ^26^ ^]^ In HNSCC, SOX2 has been demonstrated to regulate self‐renewal and tumorigenicity of stem‐like cells.^[^ ^27^ ^]^ Our previous study has shown that most of the BMI1^+^ CSCs highly expressed SOX2, but SOX2^+^ tumor cells have a more broad expression pattern than BMI1^+^ CSCS, indicating that SOX2^+^ cell might represent CSCs and progenitors.^[^ ^14^ ^]^ In this study, the SOX2^+^ cells were enriched upon PD1 blockade treatment. It suggests that SOX2^+^ CSC‐like tumor cells in HNSCC might be intrinsically resistant to CD8^+^ T cell killing.

We demonstrated that circFAT1 is critical for sustaining HNSCC CSCs self‐renewal and tumorigenicity using nude mice. For decades, CSCs were mainly investigated in the immunodeficient mouse model, which hampered the elucidation of the relationship of CSCs with immune cells and immunotherapy. Growing evidence have shown that various interactions of CSCs with the tumor immune microenvironment result in evasion of CSCs detection,^[^ ^28^ ^]^ and CSCs could directly mediate tumor resistance to adoptive cytotoxic T cell transfer‐based immunotherapy.^[^ ^29^ ^]^ These results suggest that targeting CSCs by circRNAs may be an effective candidate for corporate approaches to immunotherapy in HNSCC. As expected, by using the HNSCC model in immune competent C57Bl/6 mice, we demonstrated that circFat1 KD inhibited the SOX2^+^ cells, and the combination treatment of anti‐PD1 and circFat1 KD exhibited the most dramatic inhibition of SOX2^+^ cells.

STAT3 is abnormally activated in multiple human solid tumors, including HNSCC.^[^ ^30^ ^]^ STAT3 has long been considered to be one of the most important therapeutic targets for HNSCC.^[^ ^31^ ^]^ Our studies identify a new molecular mechanism that regulate STAT3 activation at a post‐translational level through circFAT1. HNSCC has an immunosuppressive tumor microenvironment with low tumor‐infiltrating lymphocytes.^[^ ^3^ ^]^ The abnormal activation of STAT3 might be responsible for tumor immune evasion during HNSCC invasive growth and metastasis. STAT3 might inhibit antitumor immunity of tumor cells by suppressing type 1 IFN response via multiple mechanisms.^[^ ^20^ ^]^ STAT3 can sequester STAT1 and prevent it from forming functional homodimers. Also, STAT3 can induce a suppressor to inhibit the IFN‐stimulated signaling pathways. Dephosphorylation of STAT3 by protein phosphatases plays a major role in regulating STAT3 activation. Multiple protein tyrosine phosphatases, such as MEG2,^[^ ^32^ ^]^ SHP1/2, and T‐cell Protein Tyrosine Phosphatases,^[^ ^33^ ^]^ have been shown to dephosphorylate STAT3. In our study, circFAT1 mediated its effects by binding directly to STAT3 in the cytoplasm and preventing STAT3 dephosphorylation by SHP1, leading to an increase in STAT3 phosphorylation. While circFat1 KD significantly inhibited tumor growth, we also observed that circFat1 KD significantly increased CD8^+^ T cell infiltration in tumor tissues. Given that circFat1 KD inhibited STAT3 activation, our study suggests that STAT3 plays a critical role in immune evasion. In addition to STAT3, SOX2 and KLF4 are associated with HNSCC development.^[^ ^5^ ^]^ We found that circFat1 KD also reduced the expression of SOX2 and KLF4. Because SOX2 and KLF4 are translated in cytoplasm, we cannot exclude the possibility that circFAT1 may regulate their protein levels directly.

Moreover, growing evidence suggests that the tumor resistance to PD1 blockade immunotherapy is probably due to lack of CD8^+^ T cell infiltration into tumor microenvironment after treatment.^[^ ^34^ ^]^ The response rate of HNSCC to PD1 blockade is around 20–30%, and the majority of HNSCC were irresponsive to PD1 blockade,^[^ ^4^ ^]^ suggesting that HNSCC might have an intrinsic mechanism against immunotherapy. However, the underlying mechanisms of immunotherapy resistance are poorly understood. Several molecular pathways involving WNT/*β*‐catenin, MYC, PTEN, and LKB1 have been found to inhibit antitumor immunity. Interestingly, these pathways also play an important role in maintaining cancer stemness. Very recently, we showed that the inhibition of BMI1 proteins not only helped to eliminate cancer stem cells, but also activated tumor‐intrinsic immune responses by stimulating STING‐IRF3 signaling.^[^ ^6b^ ^]^ MOC1 cells were derived from 4NQO‐induced HNSCC through multiple passaging in vitro. Compared with unperturbed tumor, xenon‐grafted MOC1 tumor had a slightly better response upon PD1 blockade treatment which was probably due to multiple passages in vitro. However, we found that circFAT1 KD did significantly improve PD1 blockade treatment by recruiting more CD8^+^ T cells. Our work showed that increasing circFAT1 in HNSCC is an important molecular mechanism which mediates the tumor immunosuppressive environment in HNSCC. Taken together, our results have important implications in developing a new combination treatment for advanced cancer by targeting circFAT1 to eliminate CSCs and to activate tumor cell‐intrinsic immune responses.

## 4 Experimental Section

##### Patients and Clinical Samples

To generate the HNSCC PDX, the discarded human HNSCC tissues were obtained without patient information from the UCLA Translational Pathological Core and were approved by the UCLA Institutional Review Board (IRB#10‐001711‐CR‐00011). The tumor tissues were sliced into small pieces and implanted into the flanks of 6‐week‐old NOD‐SCID mice as described previously.^[^ ^14^ ^]^ A total of 100 HNSCC samples from the Peking University Hospital of Stomatology (from September 2010 to October 2014) were utilized in this study. HNSCC tumor tissues and the paired normal tissues were snap‐frozen in liquid nitrogen and then stored at −80 °C until use. The inclusion criteria were 1) the tumor was located in the tongue, 2) no distant metastasis, 3) the patients received removal of the primary carcinoma and neck dissection without preoperative radiotherapy or chemotherapy, and 4) the patients were followed‐up for at least 5 years postoperation. For the 40 case‐control HNSCC samples among the 100 samples, tumor size and clinical stage were classified according to the clinical TNM staging system without pathological analysis: 1) tumor size limited in T2 and T3, 2) clinical negative cervical lymph node (cN0), and 3) no distant metastasis (M0). These 40 patients were divided into the lymph node negative and positive group according to the histopathologic examination of lymph nodes. All the surgical samples were confirmed by the pathologists using H&E‐stained sections. These experiments were approved by the Institutional Ethics Committee of Peking University School of Stomatology and all samples were obtained from patients who signed informed consent forms approving the use of their tissues for research purposes after surgery.

##### RNA FISH

The FISH assay was performed using Cy3‐labeled circFAT1 probe (5′‐Cy3‐CTTAACTGTCGGGAATCTGTCTCTTCACCTAT‐3′). Briefly, SCC23 and SCC1 cells grown on a glass coverslip were fixed in 70% ethanol for 20 min and permeabilized with 0.1% Triton X‐100 for 5 min. The cells were pretreated with prehybridization buffer (2 × saline‐sodium citrate, SSC) containing 10% formamide, and then were hybridized in 200 µL hybridization buffer (40% formamide, 10% Dextran sulfate, 1 × Denhardt's solution, 4 × SSC, 10 × 10^−3^ m DDT, 100 µg mL^−1^ yeast transfer RNA, 100 µg mL^−1^ sheared salmon sperm DNA) with 5 µL of Cy3‐labeled circFAT1 probes (20 × 10^−6^ m) at 42 °C overnight. After washing, the nucleus was stained with 4′6′‐diamidino‐2‐phenilindole (DAPI; Sigma‐Aldrich Cat#D9542), and mounted with SlowFade Antifade Reagents (Thermo Fisher Scientific Cat#S36937). For colocalization analysis of circFAT1 and STAT3, after hybridized with circFAT1 probe, the cells were incubated with anti‐STAT3 antibodies (Cell Signaling Technology, Cat#9139) at 4 °C overnight and then detected with the specific secondary antibodies. The images were taken using a Leica SP5X laser scanning confocal microscope. The colocalization for circFAT1 and STAT3 and fluorescent intensity were analyzed in cells using LAS X software (Leica, IL, USA). The foci of circFAT1 and STAT3 were counted and averaged from a total of 50 cells. circFAT1 foci were divided into two groups based on its colocalization or non‐colocalization with STAT3.

##### Nuclear and Cytoplasmic Fraction Isolation

Cytoplasmic and nuclear RNA was extracted using Thermo Fisher BioReagents (Thermo Fisher Scientific, Cat#AM1921) according to the manufacturer's instructions. Briefly, the cells were suspended and lysed with cell fraction buffer and then centrifuged at low speed to separate the nuclear fraction from the cytoplasmic fraction. Subsequently, the cytoplasmic fraction was carefully aspirated away from the nuclear pellet, and the cell disruption buffer was added to the nuclear pellet. The samples were split for RNA isolation with TRIzol (Thermo Fisher Scientific, Cat# 15596026).

##### qRT‐PCR and ChIP‐qPCR

Total RNA was isolated from tumor and normal tissue samples by using TRIzol reagent according to the manufacturer's instructions (Thermo Fisher Scientific, Cat#15596026). 1–2 µg of RNA was used for the RT reaction with random primers (Thermo Fisher Scientific, Cat#48190011) or oligo (dT) primers (Thermo Fisher Scientific Cat#18418012), dNTP mix (Thermo Fisher Scientific, Cat#18427013), and M‐MuLV Reverse Transcriptase (New England Biolabs, Cat#M0253L). The levels of mRNA were quantified using a SYBRGreen supermix (Bio‐Rad, Cat#1708880) with *GAPDH* as an internal control. The primer sequences used for qRT‐PCR are listed in Table S3 (Supporting Information). For RNase R treatment, 2 µg of RNA from SCC23 or SCC1 cells were incubated for 30 min at 37 °C with or without 5 U µg^−1^ RNase R (Epicentre Technologies, Cat#RNR07250), and subsequently purified by RNeasy MinElute Cleaning Kit (Qiagen, Cat#74204), then analyzed by qRT‐PCR. ChIP‐qPCR assays were conducted as previously described.^[^ ^6b^ ^]^ Briefly, SCC23 cells were treated with dimethyl 3,3′‐dithiobispropionimidate‐HCl (DTBP; Thermo Fisher Scientific, Cat#20665) solution and formaldehyde, and then lysed and sonicated to generate 200–500 bp DNA fragments. The fragmented chromatins were precleared and incubated with anti‐STAT1 (Cell Signaling Technology, Cat#14994) overnight at 4 °C. The precipitated DNA‐chromatin products were quantified by qPCR. Data are presented as the percentage of input DNA. The primer sequences used for ChIP‐qPCR were listed in Table S3 (Supporting Information).

##### Cell Culture and siRNA or shRNA Knockdown

Human HNSCC cell lines SCC23 and SCC1 were from the University of Michigan,^[^ ^35^ ^]^ and were maintained in DMEM containing 10% FBS and antibiotics (streptomycin and penicillin). The mouse MOC1 cell line was kindly provided by Dr. Gutkind at The University of California San Diego, and maintained in keratinocyte serum free medium containing 10% FBS. All these cells were maintained at 37 °C in 5% CO2 atmosphere. For transient transfection, 10 × 10^−9^ m siRNAs were transfected with Lipofectamine RNAiMAX reagent (Thermo Fisher Scientific, Cat#13778150) following the manufacturer's instructions. The siRNA sequences for targeting circFAT1 are 5′‐CTGTCGGGAATCTGT CTCT‐3′ (siCFAT1‐1) and 5′‐GGAATCTGTCTCTTCACCT‐3′ (siCFAT1‐2). The siRNA scramble control sequence is 5′‐TTCTCCGAACGTGTCACG T‐3′. To generate lentiviruses, the scramble control (shCtrl, Addgene, Cat#1864) or the specific shRNA lentiviral plasmids were cotransfected into HEK293T cells with two helper plasmids psPAX2 (Addgene, Cat#12260) and pMD2.G (Addgene, Cat#12259). Viral supernatants were harvested and stored at −80 °C freezer for cell infection 72 h after transfection. Cells were infected with lentiviruses in the presence of polybrene (Sigma‐Aldrich, Cat#H9268), selected with puromycin (Sigma‐Aldrich, Cat#P9620) at 1 µg mL^−1^ for 5 days and expanded before being used for subsequent assays. The shRNA sequence for human circFAT1 is 5′‐CTGTCGGGAATCTGTCTCT‐3′, and the shRNA sequence for mouse circFat1 is 5′‐TGGCCACCTGTTTCTTCAT‐3′. The STAT3 shRNA lentiviral plasmid (shSTAT3) was purchased from Sigma (Sigma‐Aldrich, Cat#NM003150).

##### Cell Proliferation Assay and Matrigel Invasion Assays

The effects of circFAT1 KD on the proliferation of SCC23 and SCC1 cells were determined using the MTT Cell Proliferation Assay Kit (Thermo Fisher Scientific, Cat#M6494). The cells were plated into 96‐well plates (2 × 10^3^ cells per well). After transfection with siRNAs at different time points, MTT solvent were added, and the absorbance at 450 nm was measured using a microplate spectrophotometer (Bio‐Tek Instruments Inc, Winosski, VT). For Matrigel invasion assays, transwell inserts with 8 µm pore size membrane coated with Matrigel (Corning, Cat#354480) were placed in the wells of 24‐well culture plates. DMEM containing 10% FBS (600 µL) was added to the lower chamber. Cells were resuspended in 100 µL serum‐free DMEM (1 × 10^5^ cells) and added to the upper chamber. After 24 h of incubation at 37 °C with 5% CO_2_, cells on the top side of the membranes were manually removed with a cotton swab. The membranes were fixed in 70% ethanol and stained with 1% crystal violet in 20% ethanol. After 10 min of incubation, the membranes were washed thoroughly in water and images were captured. The number of invasive cells were randomly counted from three fields and averaged.

##### CSCs Isolation and Tumorsphere Formation Assays

For isolation of CSCs from SCC23 and SCC1 cells, ALDH^high^ CSCs were sorted with an ALDHEFLUOR assay kit (STEMCELL Technologies, Cat#01700). As ALDH^low^ controls, the ALDEFLUOR‐stained cells were treated with a specific ALDH inhibitor DEAB. ALDH^high^ and ALDH^low^ subpopulations were separated by a fluorescence‐activated cell sorting (FACS) at the flow core of the UCLA Jonsson Comprehensive cancer center. For isolating CSCs from human HNSCC PDX, human HNSCC xenografted tumors were chopped and then digested into single cell suspensions by using a human tumor cell dissociation kit (Miltenyi Biotec, Cat#130095929). The EpCAM^+^ tumor cells were isolated using EpCAM MicroBeads (Miltenyi Biotec, Cat#130061101), and the ALDH^high^ and ALDH^low^ subpopulations from the EpCAM^+^ tumor cells were then sorted with an ALDHEFLUOR assay kit. For tumorsphere formation assays, FACS‐sorted cells were seeded in ultralow attachment plates and cultured in serum‐free DMEM/F12 (Thermo Fisher Scientific, Cat#11330‐032) with 1% B27 supplement (Thermo Fisher Scientific, Cat#17504044), 1% N2 supplement (Thermo Fisher Scientific, Cat#17502048), human recombinant epidermal growth factor (EGF, 20 ng mL^−1^; R&D Systems, Cat#236‐EG‐01M), and human recombinant basic fibroblast growth factor (bFGF, 10 ng mL^−1^; R&D Systems, Cat#233‐FB‐025/CF), in a humidified 5% CO_2_ incubator at 37 °C. Spheres with a diameter over 40 µm were counted under the microscope after 2 weeks.

##### In Vivo Tumor Growth in Mice

Female Nude/SCID mice at 6–8 weeks were purchased from The Jackson Laboratory. All mouse studies were performed strictly according to the animal protocol #2007‐062‐41 approved by UCLA Animal Research Committee. SCC23 cells (1 × 10^6^) with or without circFat1 KD were injected subcutaneously into the flank area of nude mice. The tumor growth was monitored every day, and the tumor size was measured every week. After 4 weeks, the mice were sacrificed and tumors were weighed. For vivo limiting‐dilution assay, ALDH^high^ CSCs from SCC23 cells or EpCAM^+^ALDH^high^ CSCs were infected with the lentiviruses expressing shCFAT1 or shCtrl for 24 h. After rapid selection with puromycin, different numbers of cells were mixed with an equal volume of Matrigel and injected subcutaneously into the flank area of nude mice for 4 weeks. The data was analyzed using the Extreme Limiting Dilution Analysis software (http://bioinf.wehi.edu.au/software/elda/). For orthotopical tumor formation, MOC1 cells (1 × 10^6^) with or without circFat1 KD were injected in the tongue of C57BL/6J mice and grown for 4 weeks. For anti‐PD1 antibody treatment, tumor‐bearing mice were given anti‐PD1 antibodies (BioXcell, Cat#BE0146, 200 µg per mouse) or InVivoPlus rat IgG2a isotype control (BioXcell, Cat#BP0089, 200 µg per mouse) twice a week for 4 weeks. Mice were sacrificed, and the tongues and cervical lymph nodes were dissected and isolated immediately. Tumor volume was determined using the volume formula for an ellipsoid: 1/2 × *D* × *d* ^2^ where *D* is the longer diameter and *d* is the shorter diameter. To examine cervical lymph node metastasis of HNSCC, the sections of cervical lymph nodes were immunostained with anti‐PCK antibodies.

##### RNA‐Seq and Pathway Enrichment Analysis

Total RNAs were isolated from SCC23 cells with shCFAT1 or shCtrl using a RNeasy Micro Kit (QIAGEN, Cat#74004), and RNA quality was validated using an Agilent 2100 Bioanalyzer. Library constructions with the KAPA RNA‐Seq Library Preparation Kits (KAPA Biosystems, Cat#07960140001) was done at the UCLA sequencing core, and RNAs were single‐end sequenced on Illumina HiSeq 3000 machines. The online DAVID (https://david.ncifcrf.gov/summary.jsp) bioinformatics resources were applied to examine the differentially expressed genes under the category of KEGG_PATHWAY. The heatmap was made by GraphPad Prism 8.0 (GraphPad software, Inc.). Ranked lists of log2 fold change were analyzed using GSEA by the Broad Institute Data Packages.^[^ ^36^ ^]^ The raw data are deposited at the GEO under the subseries entry GSE150398.

##### RIP and RNA Pull‐Down Assays

RIP assays were carried out by using the Magna RIP RNA‐Binding Protein Immunoprecipitation Kit (Millipore, Bedford, MA). In brief, magnetic beads were incubated with anti‐STAT3 (Cell Signaling Technology, Cat#9139), anti‐Flag (Sigma‐Aldrich, cat#F1804), and IgG control. The SCC23 cells were collected and lysed in RIP Lysis buffer. The lysates were immunoprecipitated with beads coated with anti‐STAT3, anti‐SHP1 or IgG. The coprecipitated RNAs were eluted in the aqueous solution and then measured using qRT‐PCR with a specific primer set. The results were normalized relative to the input control. For RNA pull‐down assays, 1 × 10^7^ cells were lysed in 500 µL CelLytic M buffer (Sigma‐Aldrich, Cat#C2978), and incubated with 3 µg of biotinylated DNA oligo probes against endogenous circFAT1 at 4 °C overnight. A total of 40 µL of streptavidin‐coated magnetic beads (Thermo Fisher Scientific Cat#88816) were added to each binding reaction and further incubated at room temperature for 2 h. The beads were pulled down and washed five times with CelLytic M buffer. The pull‐down proteins were resolved and reanalyzed by Western blot. The probe sequences are 5′‐biotin‐CTTAACTGTCGGGAATCTGTCTCTTCACCTAT‐3′ (circFAT1), 5′‐biotin‐TCCTTGTGGATTTCCACTTGTAATTTTGTACA‐3′ (FAT1 exon 3), and 5′‐biotin‐ ACGTAGCCGTTGCATTTGCCGTAGCCCTGTGG ‐3′ (control)

##### Western Blot Analysis, ELISA Assays, and IP

Cells were lysed using the radioimmunoprecipitation assay buffer (Sigma‐Aldrich, Cat#R0278) supplemented with a cocktail of protease inhibitors (Thermo Fisher Scientific, Cat##78430) and phosphatase inhibitors (Sigma‐Aldrich, Cat#4906845001). The whole cell protein extracts were resolved on a 10% SDS polyacrylamide gel and then transferred to a polyvinylidene difluoride membrane. Membranes were blocked with 5% milk for 1 h and incubated with primary antibodies at 4 °C overnight. Primary antibodies used in this study were: anti‐pSTAT3 (Tyr705) (1: 1000; Cell Signaling Technology, Cat # 9145), anti‐STAT3 (1: 1000; Cell Signaling Technology, Cat#9139), anti‐SOX2 (1:1000; Cell Signaling Technology, Cat#14962), anti‐SHP1 (1: 1000; Cell Signaling Technology, Cat#3759), anti‐FAT1 (1:2000; Sigma‐Aldrich, Cat#HPA023882), and *anti‐GAPDH* (1:1000; Cell Signaling Technology, Cat# 5174). To measure the protein levels of CXCL9 and CXCL10 produced by cells, SCC23 and SCC1 cells transfected with shCFAT1 or shCtrl were cultured for 48 h. The supernatants were collected and measured using ELISA (R&D Systems, Cat# DCX900 and DIP100) according to the manufacturer's instructions. For IP assays, SCC23 cells (1 × 10^7^) were lysed in 1 mL of CelLytic M buffer (Sigma‐Aldrich, Cat#C2978) for 20 min on ice, and the supernatant was collected after centrifugation at 12 000 × *g* at 4 °C. Subsequently, the supernatants were incubated with 4 µL of anti‐STAT3 (Cell Signaling Technology, Cat#9139) or anti‐SHP1 antibodies (Cell Signaling Technology, Cat#3759) at 4 °C overnight. The immunocomplexes were pulled down by incubation with a 40 µL of Magna ChIP Protein A+G Magnetic Beads (Millipore, Cat#16663) for an additional 2 h. Immunoprecipitates were washed three times with PBS with 0.1% NP40 buffer at 4 °C, and proteins bound to the beads were eluted with SDS‐loading buffer at 98 °C for 5 min and examined by Western blot analysis.

##### Immunostaining

Tumor samples were sectioned at 5 µm and processed as previously described.^[^ ^14^ ^]^ For immunofluorescent staining, the sections were stained with the following primary antibodies: anti‐PCK (Abcam Cat#ab9377; 1:200), anti‐active‐caspase3 (Cell Signaling Technology, Cat#9661; 1:200), anti‐CD8 (Cell Signaling Technology Cat#98941; 1:200), anti‐Granzyme B (R&D Systems Cat#AF1865; 1:100), and anti‐CXCL10 (R&D Systems Cat#MAB466; 1:100). The signals were detected using secondary antibodies conjugated with the fluorescent marker Cy2 or Cy3 (Jackson ImmunoResearch Laboratories). Sections were then stained with 4′6′‐diamidino‐2‐phenilindole (DAPI; Sigma‐Aldrich Cat#D9542) and mounted with SlowFade Antifade Reagents (Thermo Fisher Scientific Cat#S36937). For immunohistochemistry, sections were stained with anti‐SOX2 antibodies (Millipore, Cat#AB5603; 1:100) anti‐pSTAT3 (Tyr705) (Cell Signaling Technology, Cat # 9145; 1:100) at 4 °C overnight and then incubated with horseradish perioxidase‐labeled polymer for 60 min. The signals were detected with AEC+ chromogen (Dako EnVision System Cat#MP‐6401‐15), and counter‐stained with hematoxylin.

##### Statistical Analyses

Statistical parameters of the analyses are reported in the figure legends. Statistical analyses were performed using GraphPad Prism 8.0 for windows (GraphPad software, Inc.). Two tailed Student's *t‐*test was performed between two groups. The survival rates were calculated using Kaplan–Meier method and analyzed by log‐rank test. The differences between HNSCC cells transfected with siCtrl, siCFAT‐1, and siCFAT1‐2 were determined by one‐way ANOVA followed by the Tukey's HSD post hoc tests to minimize type I errors. All in vitro experiments were repeated at least twice. To compare the treatment efficiency of anti‐PD1 in MOC1 derived tumors with circFat1 KD or not, the differences were assessed using two‐way ANOVA. *p* < 0.05 was considered significant.

## Conflict of Interest

The authors declare no conflict of interest.

## Author Contributions

L.J. and Y.W. performed the experiments. L.J. and C.‐Y.W. designed the experiments and wrote the manuscript.

## Supporting information



Supporting Information
