# Supplementary material for: circFAT1 Promotes Cancer Stemness and Immune Evasion by Promoting STAT3 Activation
Source: Adv Sci (Weinh). 2021 May 2;8(13):2003376. doi: 10.1002/advs.202003376 (PMC8261519; doi:10.1002/advs.202003376)
Supplement: Supplementary file 1 — Supporting Information [file ADVS-8-2003376-s001.pdf]

© 2020 Wiley-VCH GmbH

ADVANCED SCIENCE Open Access

Supporting Information

for *Adv. Sci.*, DOI: 10.1002/advs.202003376

circFAT1 Promotes Cancer Stemness and Immune Evasion by Promoting STAT3 Activation

*Lingfei Jia, Yilun Wang, and Cun-Yu Wang**

circFAT1 Promotes Cancer Stemness and Immune Evasion by Promoting STAT3 Activation

Lingfei Jia, Yilun Wang, and Cun-Yu Wang[*]

L. Jia, Y. Wang, Pro. C.-Y. Wang

Jonsson Comprehensive Cancer Center, UCLA,

Los Angeles, CA 90095, USA.

E-mail: cwang@dentistry.ucla.edu

L. Jia, Y. Wang, Pro. C.-Y. Wang

Laboratory of Molecular Signaling, Division of Oral Biology and Medicine, School of Dentistry, UCLA,

Los Angeles, CA 90095, USA.

Pro. C.-Y. Wang

Department of Bioengineering, Henry Samueli School of Engineering and Applied Science, UCLA,

Los Angeles, CA 90095, USA

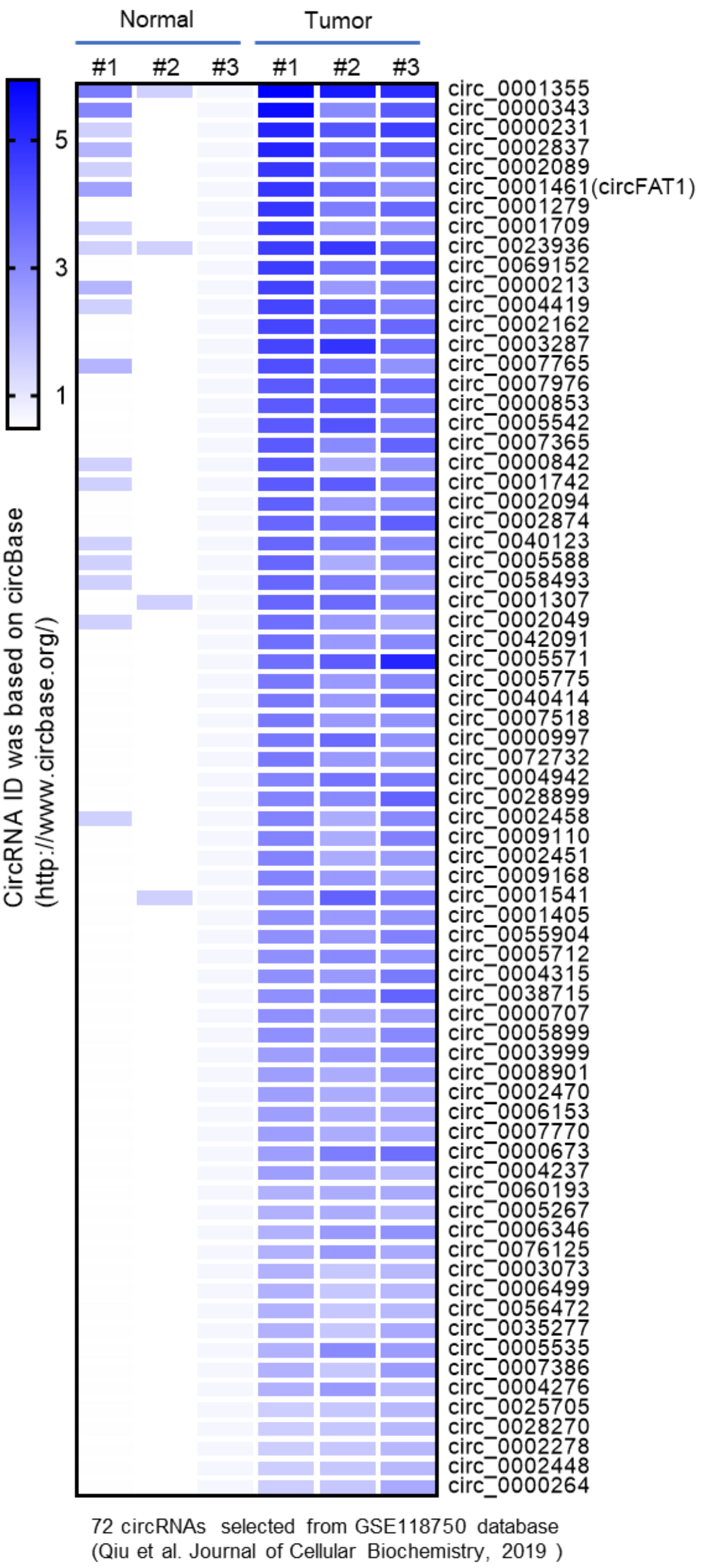

Figure S1. The cluster heat map showed the most upregulated 72 circRNAs in HNSCC tissues compared with adjacent normal tissues.

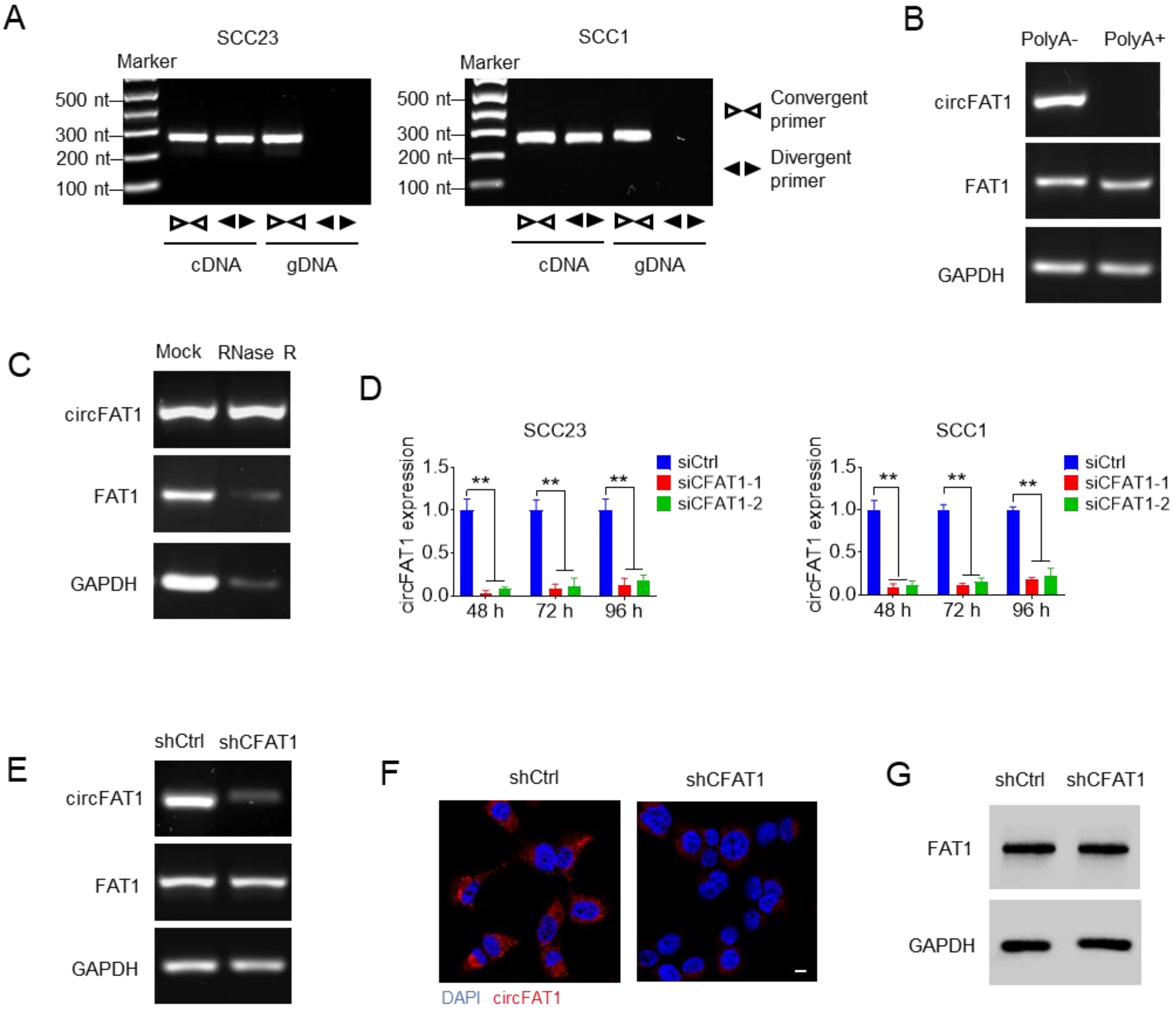

Figure S2. The characteristics of circFAT1**.** A) qRT-PCR products with divergent primers showing circularization of human circFAT1 in cDNA, but not in genomic DNA. cDNA, complementary DNA. gDNA, genomic DNA. B) RT-PCR products of *circFAT1*, *FAT1* and *GAPDH* from the cDNA synthesized by using random or oligo (dT) primer. PolyA- and PolyA+ represented non-poly(A)-tailed, and poly(A)-tailed RNAs respectively. C) RT-PCR products of *circFAT1*, *FAT1* and *GAPDH* after Rnase R treatment. D) qRT-PCR analysis of *circFAT1* and *FAT1* expression in SCC23 and SCC1 cells treated with siRNA control (siCtrl), siRNA circFAT1-1 (siCFAT1-1) and siRNA circFAT-2 (siCFAT1-2) for 48 h, 72 h, and 96 h.

Means ± SD are shown. **p < 0.01 by unpaired Student's *t* test. E) RT-PCR products of *circFAT1*, *FAT1* and *GAPDH* in SCC23 cells after stable transfection of shRNA circFAT1 (shCFAT1). F) Identification of circFAT1 expression by FISH in SCC23 after stable transfection of shCFAT1. circFAT1 probe was labeled with Cy3 (red). Nuclei was stained with DAPI (blue). Scale bar, 10 μm. G) Western blot analysis of FAT1 in in SCC23 after stable transfection of shCFAT1.

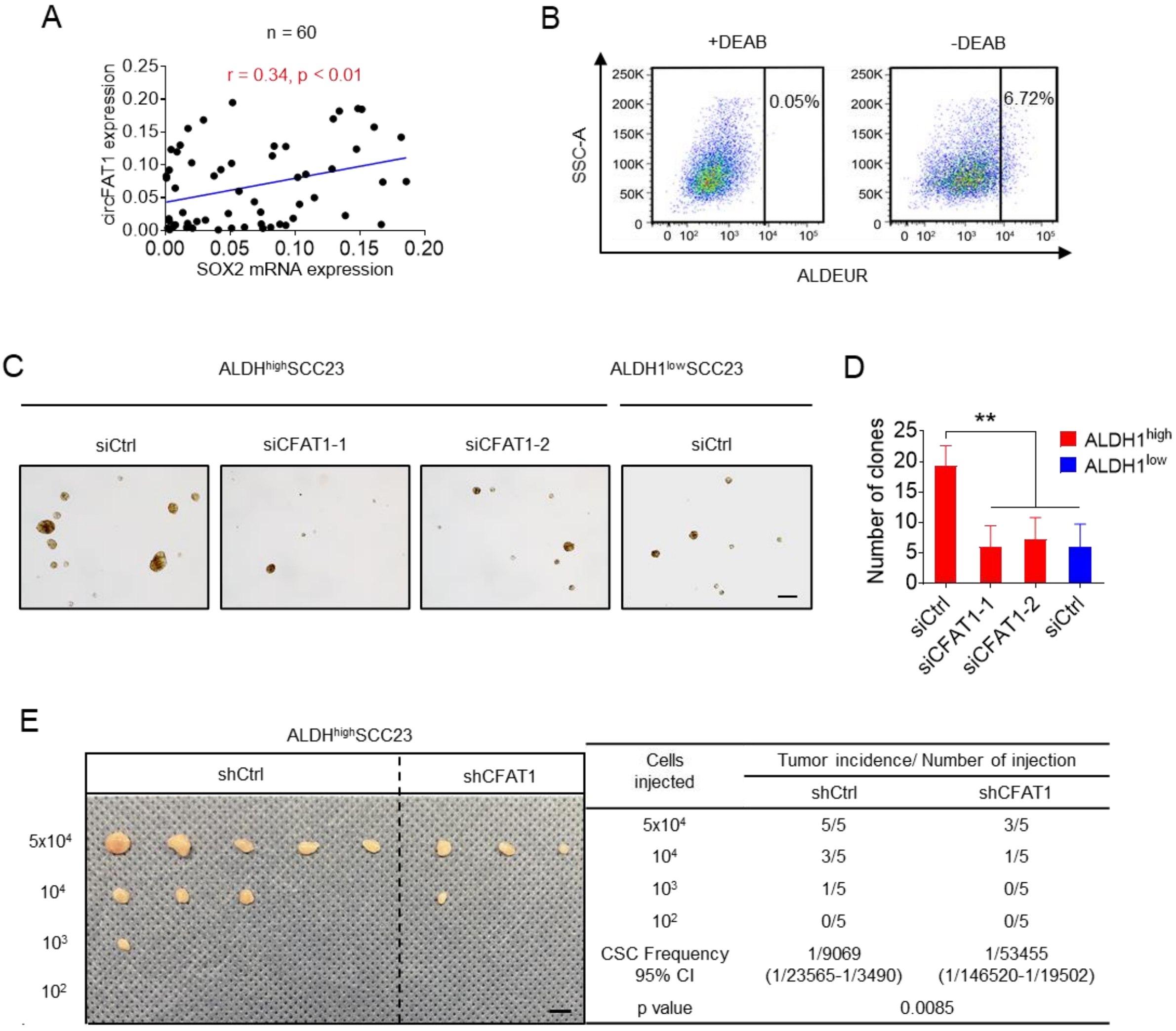

Figure S3. circFAT1 KD significantly inhibits the tumorigenic ability of ALDH^{high} CSCs. A）Positive correlation between *SOX2* and *circFAT1* mRNA expression in 60 HNSCC samples. Statistical analysis was performed using Pearson’s correlation coefficient analysis, with r and p values as indicated. B) Representative fluorescence-activated cell sorting (FACS) plot of ALDH^{high} CSCs and ALDH^{low} non-CSCs isolated from tumor cells. ALDH^{high} CSCs were sorted with an ALDHEFLUOR assay kit (ALDUOR). As a control, the ALDUOR-stained cells were treated with a specific ALDH inhibitor diethylaminobenzaldehyde (DEAB). C) Representative tumorspheres formation of ALDH^{low} SCC23 cells and ALDH^{high} SCC23 cells

transfected with siRNAs as indicated. Scale bar, 100 µm. D) Quantification of number of tumorspheres from ALDH^{low} SCC23 cells and ALDH^{high} SCC23 cells transfected with siRNAs as indicated. Means ± SD are shown. **p < 0.01 by unpaired Student's *t* test. E) *In vivo* limiting dilution analysis of ALDH^{high} SCC23 cells transduced with shCtrl and shCFAT1 (n = 5). The frequency of tumor formation at each cell dose injected is shown. The data were analyzed using ELDA software. Scale bar, 1 cm.

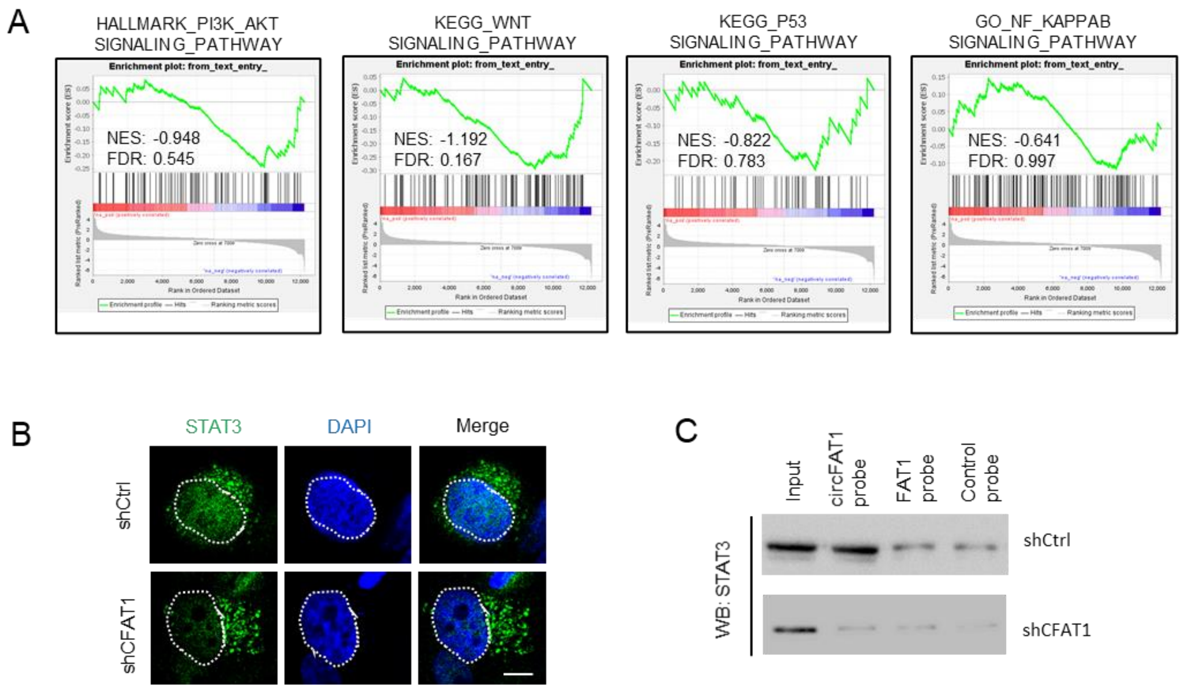

Figure S4. GSEA analysis of signaling pathways related with cancer stemness. A) GSEA analysis in circFAT1-regulated genes in SCC23 cells. The colored panel at the bottom indicates the level of differential expression from the most up-regulated (red) to the most down-regulated (blue). B) Immunofluorescence detection of nuclear translocation of STAT3 (green) in SCC23 transduced with shCFAT1. Nuclei was stained with DAPI (blue). White dashed lines demark nuclei region. Scale bar, 10 μm. C) RNA pull-down assay detected the interactions between circFAT1 and STAT3 by Western blot (WB) in SCC23 transduced with shCFAT1.

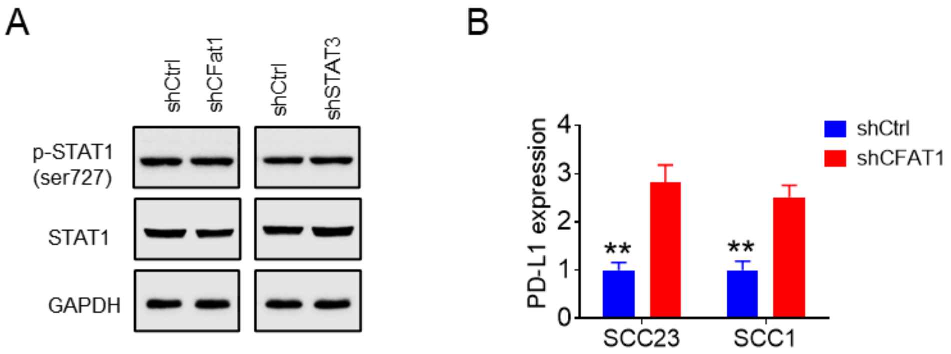

Figure S5. circFAT1 KD does not affect the levels of STAT1 and pSTAT1. A) Western blot analysis of the pSTAT1 and STAT1 in SCC23 cells with circFAT1 KD. B) qRT-PCR analysis of *PD-L1* in SCC23 and SCC1 cells with circFAT1 KD. Means ± SD are shown. $^{**}p < 0.01$ by unpaired Student's *t* test.

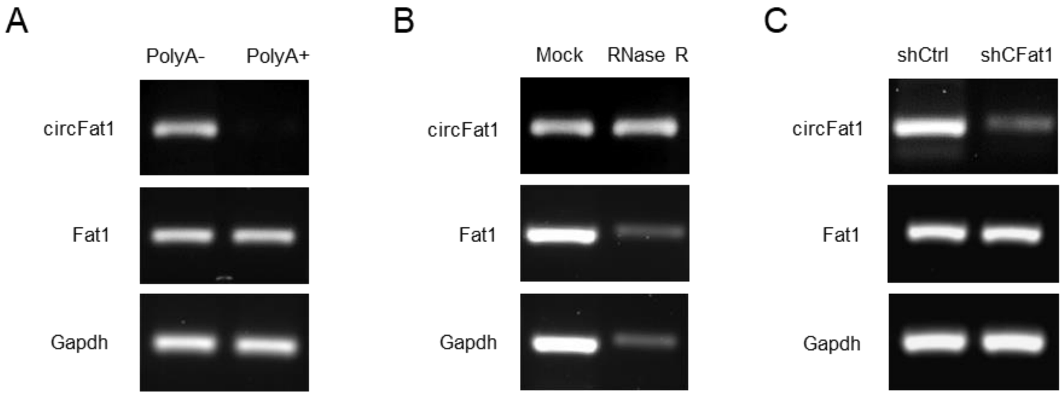

Figure S6. The characteristics of mouse circFat1**.** A) RT-PCR products of *circFat1*, *Fat1* and *Gapdh* from the cDNA synthesized by using random or oligo (dT) primer. PolyA- and PolyA+ represented non-poly(A)-tailed and poly(A)-tailed RNAs, respectively. B) RT-PCR products of *circFat1*, *Fat1* and *Gapdh* after RNase R treatment. C) RT-PCR products of *circFat1*, *Fat1*, and *Gapdh* in MOC1 cells transduced with shRNA circFat1 (shCFat1).

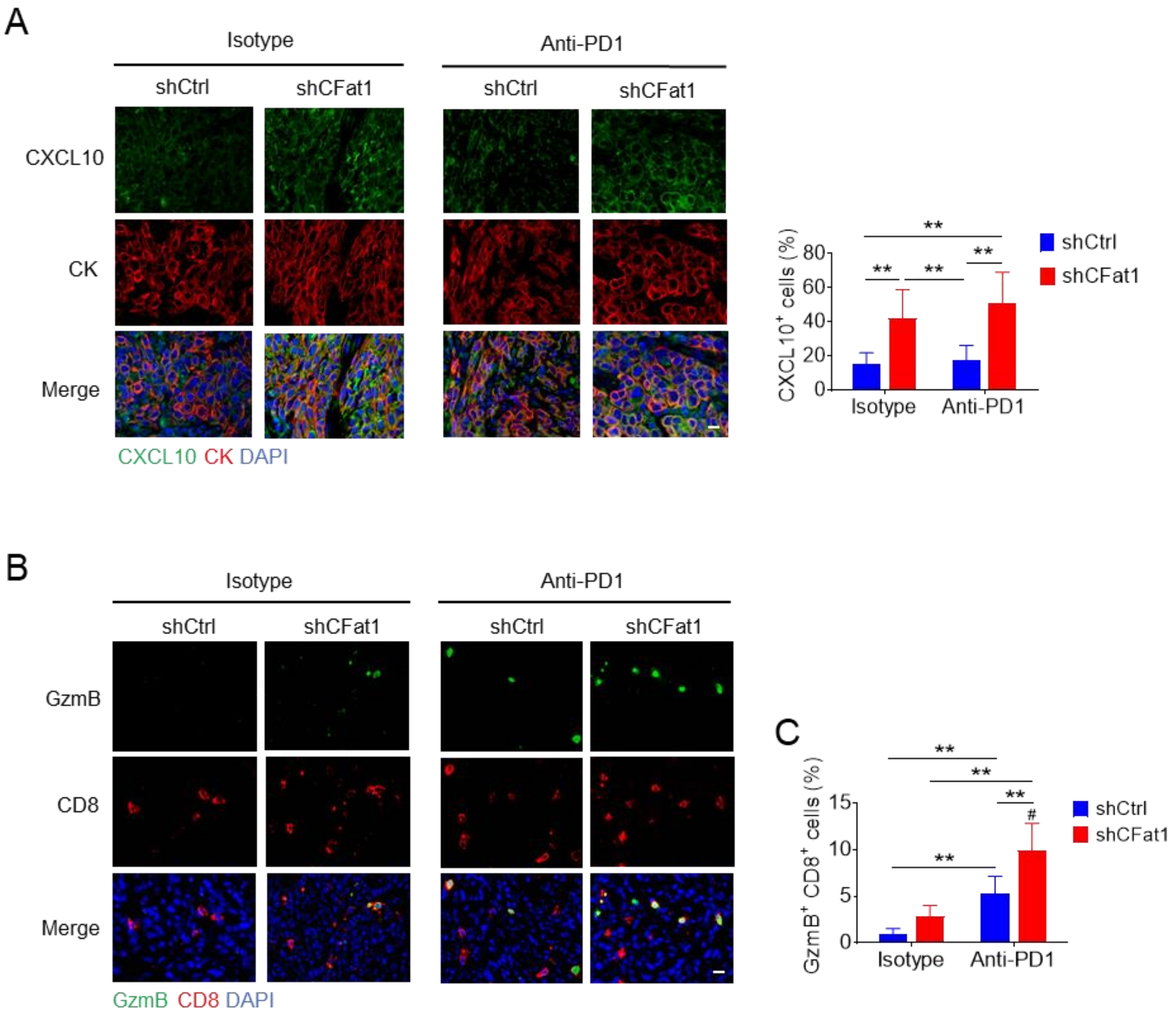

Figure S7. circFat1 KD potentiates PD-1 blockade immunotherapy. A) Immunofluorescent staining and quantification of CXCL10 in HNSCC upon anti-PD1 treatment. CK, Pan-cytokeratin. Means ± SD are shown (n = 8). **p < 0.01 by two-way ANOVA. B) Immunofluorescent staining of $CD8^+$ and Granzyme B $(GzmB)^+$ T cells. The upper, middle and lower panels respectively show the staining of GzmB (green), CD8 (Red), and CD8/GzmB co-localization. Nuclei were visualized by DAPI (Blue). Scale bar, 10 μm. C) Quantifications of percentage of $GzmB^+CD8^+$ T cells in tumor. Means ± SD are shown (n = 8). **p < 0.01 by two-way ANOVA. #p < 0.05 treatment x genotype interaction.

Table S1. The clinical features of HNSCC patients with positive and negative lymph node metastases.

Lymph node Negative (pathological)						Lymph node Positive (pathological)					
NO.	Age/ gender	Clinical TNM classification	Differentiation	Final outcome	Follow-up period (yr)	NO.	Age/ gender	Clinical TNM classification	Differentiation	Final outcome	Follow-up period (yr)
1	40/M	$T_3N_0M_0$	Moderate	Alive	6	21	41/M	$T_3N_0M_0$	Moderate	Dead	2.5
2	53/M	$T_3N_0M_0$	Moderate	Alive	6	22	58/M	$T_3N_0M_0$	Moderate	Dead	0.5
3	42/M	$T_3N_0M_0$	Moderate	Alive	7	23	46/M	$T_3N_0M_0$	Moderate	Dead	3
4	40/F	$T_3N_0M_0$	Moderate	Alive	7.5	24	56/F	$T_3N_0M_0$	Moderate	Dead	3
5	51/M	$T_3N_0M_0$	Moderate	Alive	7	25	50/M	$T_3N_0M_0$	Moderate	Dead	2.5
6	66/M	$T_3N_0M_0$	Moderate	Alive	5	26	64/M	$T_2N_0M_0$	Moderate	Dead	1
7	68/F	$T_2N_0M_0$	Moderate	Alive	5	27	67/F	$T_2N_0M_0$	Moderate	Dead	1
8	52/F	$T_2N_0M_0$	Moderate	Alive	5	28	59/F	$T_2N_0M_0$	Moderate	Dead	2
9	40/M	$T_2N_0M_0$	Moderate	Alive	6	29	43/M	$T_2N_0M_0$	Moderate	Dead	2
10	53/M	$T_2N_0M_0$	Moderate	Alive	6.5	30	53/M	$T_2N_0M_0$	Moderate	Dead	0.5
11	41/M	$T_2N_0M_0$	Moderate	Alive	6	31	43/M	$T_2N_0M_0$	Moderate	Dead	4
12	69/M	$T_2N_0M_0$	Moderate	Alive	6	32	67/M	$T_2N_0M_0$	Moderate	Dead	4
13	54/F	$T_2N_0M_0$	Moderate	Alive	6.5	33	52/F	$T_2N_0M_0$	Moderate	Dead	2.5
14	62/F	$T_2N_0M_0$	Moderate	Alive	7	34	65/F	$T_2N_0M_0$	Moderate	Dead	3
15	47/M	$T_2N_0M_0$	Moderate	Alive	6	35	48/M	$T_2N_0M_0$	Moderate	Dead	2.5
16	50/M	$T_2N_0M_0$	Moderate	Alive	6.5	36	53/M	$T_2N_0M_0$	Moderate	Dead	2.5
17	61/M	$T_2N_0M_0$	Moderate	Alive	7	37	61/M	$T_2N_0M_0$	Moderate	Dead	1.5
18	45/M	$T_2N_0M_0$	Moderate	Alive	5	38	47/M	$T_2N_0M_0$	Moderate	Dead	2
19	59/F	$T_2N_0M_0$	Moderate	Alive	5	39	60/F	$T_2N_0M_0$	Moderate	Dead	2
20	62/M	$T_2N_0M_0$	Moderate	Alive	5	40	63/M	$T_2N_0M_0$	Moderate	Dead	1.5

Abbreviations: M, male; F, female; T, Tumor size; N0, negative cervical lymph node based on clinical examination; M0, no distant metastasis.

Table S2. Alignment of Mouse Fat1 exon2 (upper line) and Human FAT1 exon2 (lower line),

Identity = 81.91% (2689/3283)

```
1      GTGGCCAGGAGTTAAAAAATGGGGAGACACTTGACCTTGCTTCTGCTTCTGCTCCTCTTC
        |  ||   ||||||  ||||||||||||| ||| | ||||| ||||||||||||||  ||
1      ATTCCCGACAGTTAAGCAATGGGGAGACATTTGGCTTTGCTCCTGCTTCTGCTCCTTCTC

61     CTCCAGCAGTTTGGAGACAGTGATGGCAGCCAAAGACTTGAACCCACCCCTCCTATCCAG
        |||| || |||||||||||||||||||||||| |||||||||  || ||||   | |||
61     TTCCAACATTTTGGAGACAGTGATGGCAGCCAACGACTTGAACAGACTCCTC...TGCAG

121    TTTACACACTTCCAGTACAATGTCACTGTGCACGAGAACTCGGCAGCGAAGACCTATGTT
       ||||||||| || ||||||| ||||| ||||| |||||||| ||||| ||||| ||||| 
118    TTTACACACCTCGAGTACAACGTCACCGTGCAGGAGAACTCTGCAGCTAAGACTTATGTG

181    GGCCACCCCAGAAAGATGGGCATCTACATCTTAGACCCCTCGTGGGAGATAAGGTACAAA
       || || ||    ||||||||   | |||||   | | ||  |||||||  ||||||||||||
178    GGGCATCCTGTCAAGATGGGTGTTTACATTACACATCCAGCGTGGGAAGTAAGGTACAAA

241    ATCGTCTCAGGAGACAGCGAAAACCTGTTCAAAGCGGAAGAGTATGTTCTCGGAGACTTC
       || || || ||||||| |||||||||||||||| ||||||||  ||||||||||||| 
238    ATTGTTTCCGGAGACAGTGAAAACCTGTTCAAAGCTGAAGAGTACATTCTCGGAGACTTT

301    TGCTTTCTAAGGATAAGAACCAAAGGAGGGAATACCGCCATCCTGAACAGAGAAGTGAGA
       ||||||||||| ||||| ||||||||||| ||||| || || || || ||||||||||  
298    TGCTTTCTAAGAATAAGGACCAAAGGAGGAAATACAGCTATTCTTAATAGAGAAGTGAAG

361    GACCATTACACGCTGATCGTCAAGGCAGTGGAGAAAGCCACCGATGCTGAGGCCCGAGCG
       || || |||||  |||| || || ||| | || |||   ||  |||  | ||| ||| | 
358    GATCACTACACATTGATAGTGAAAGCACTTGAAAAAAATACTAATGTGGAGGCGCGAACA

421    AAGGTTCGGGTACAAGTGCTGGATACAAACGACTTAAGGCCCTTATTCTCCCCCACCTCC
       |||||  |||| || |||||||||||||| ||||| || || |||||||| |||||||| 
418    AAGGTCAGGGTGCAGGTGCTGGATACAAATGACTTGAGACCGTTATTCTCACCCACCTCA

481    TACAGCGTCTCTTTGCCGGAAAACACAGCCATAAGGACCAGCATCGCAAGAGTCAGTGCC
       |||||||| ||||| || ||||||||||| ||||||||||| |||||||||||||| |||
478    TACAGCGTTTCTTTACCTGAAAACACAGCTATAAGGACCAGTATCGCAAGAGTCAGCGCC

541    ACGGATGCGGACATTGGGACCAATGGCGAATTTTACTACAGTTTTAAAGACAGAACTGAC
       |||||||| ||||| || ||||| || |||||||||||||||||||||||  |||| || 
538    ACGGATGCAGACATAGGAACCAACGGGGAATTTTACTACAGTTTTAAAGATCGAACAGAT

601    GTGTTTGCTATCCACCCGACCAGCGGCGTCGTTGTTTTGACTGGCAGGCTTGATTTCCTA
        |||||||||| ||||| ||||| || ||  | || || ||||| || ||||||| ||||
598    ATGTTTGCTATTCACCCAACCAGTGGTGTGATAGTGTTAACTGGTAGACTTGATTACCTA

661    GAGACCCAGCTCTATGAGCTGGAGATTCTGGCTGCGGACCGGGGGATGAAGCTGTACGGT
       |||||| ||||||||||| |||| || || ||||||||||| || |||||| |||| || 
658    GAGACCAAGCTCTATGAGATGGAAATCCTCGCTGCGGACCGTGGCATGAAGTTGTATGGG

721    AGCAGTGGGGTCAGCAGCCTGGCCAAGCTGACAGTTCACGTGGAGCAGGCCAACGAGTGC
       ||||||||  |||||||| ||||||||| || || ||| | || |||||||| || || 
718    AGCAGTGGCATCAGCAGCATGGCCAAGCTAACGGTGCACATCGAACAGGCCAATGAATGT
```

```
781   GCGCCCATCATAACCGCGGTGACGTTGTCGCCCTCTGAGCTGGACAAGGACCCAACCTAC
      || ||  | ||||| || ||||| ||||| || || || ||||||| ||||||| | ||
778   GCTCCGGTGATAACAGCAGTGACATTGTCACCATCAGAACTGGACAGGGACCCAGCATAT

841   GCCATCATCACCGTGGAGGACTGTGATCAGGGTGCCAACGGGGAGATAGCGTCTTTGAGC
      || ||  | || ||||| ||||| |||||||||||||| || || ||||| ||||| |||
838   GCAATTGTGACAGTGGATGACTGCGATCAGGGTGCCAATGGTGACATAGCATCTTTAAGC

901   ATCGTGGCTGGCGACCTCCTTCAGCAGTTTAAAACAGTGAGGTCTTTCCCAGGGAGTAAA
      |||||||| || ||||| || |||||||||| |||||||||||| || |||||||||||
898   ATCGTGGCAGGTGACCTTCTCCAGCAGTTTAGAACAGTGAGGTCCTTTCCAGGGAGTAAG

961   GCATTCAAAGTGAAAGCCGTCGGGGCCGTCGACTGGGACAGCCATCCTTACGGCTACAAC
      |  |  ||||| |||||| |||| | | | || |||||||| ||||||| |||||||||
958   GAGTATAAAGTCAAAGCCATCGGTGGCATTGATTGGGACAGTCATCCTTTCGGCTACAAT

1021  CTGACATTGCAGGCTAAAGACAAAGGGACTCCTCCCCAGTTTTCCCCCGTGAAAGTTGTT
      || ||| | ||||||||||| ||||| ||||| |||||||| ||  | || |||||  ||
1018  CTCACACTACAGGCTAAAGATAAAGGAACTCCGCCCCAGTTCTCTTCTGTTAAAGTCATT

1081  CACATCATTTCTCCACAGTTCAGAGCTGGCCCTGTCAAGTTTGAAATGGATGTTTACAGA
      ||| | | |||||||||||||| ||| || || ||||||||||||| |||||||||||||
1078  CACGTGACTTCTCCACAGTTCAAAGCCGGGCCAGTCAAGTTTGAAAAGGATGTTTACAGA

1141  GCCGAGATCAGTGAGTTTGCCCCTCCACATACACCCGTGGTCCTGGTCAAAGCCATTCCT
      || || || ||||| ||||| |||||  | ||||| |||||| |||| || |||||||||
1138  GCAGAAATAAGTGAATTTGCTCCTCCCAACACACCTGTGGTCATGGTAAAGGCCATTCCT

1201  AGTTATTCCCATTTGAGGTATGTTTTTAAAAGCGCTCCTGGAAAACCTAAATTCGGTTTG
        ||||||||||||||||||||||||||||||  | ||||||||| |||||||| ||||
1198  GCTTATTCCCATTTGAGGTATGTTTTTAAAAGTACACCTGGAAAAGCTAAATTCAGTTTA

1261  AATCACAACACGGGTCTCATTTCCATTTTAGAACCCATTAGAAGGCAGCACACATCCCAT
      ||| ||||||| ||||||||||| |||||||||||  ||| ||| |||||  || |||||
1258  AATTACAACACTGGTCTCATTTCTATTTTAGAACCAGTTAAAAGACAGCAGGCAGCCCAT

1321  TTTGAACTCGAGGTGACAACAAGTGACAAAAGAGCCTCCGCCCGAGTGGTGGTCAAAGTT
      |||||||| || || ||||||||||||| || ||| ||| ||   ||  |||| |||||
1318  TTTGAACTTGAAGTAACAACAAGTGACAGAAAAGCGTCCACCAAGGTCTTGGTGAAAGTC

1381  TTAGGTACAAACAGCAACCCCCCTGAGTTTACACAGACCTCGTACAAAGCATCCATCGAT
      |||||| |||| ||||| |||||||| ||||| |||||  ||||||||||  |  | |||
1378  TTAGGTGCAAATAGCAATCCCCCTGAATTTACCCAGACAGCGTACAAAGCTGCTTTTGAT

1441  GAGAACGCGCCTATCGGTGCCGCAGTCACGAGGGTGAGTGCGATGGACCCCGACGAGGGG
      ||||||| ||| || ||| |  | |||| |||  |||||||  | ||||| || |||||
1438  GAGAACGTGCCCATTGGTACTACTGTCATGAGCCTGAGTGCCGTAGACCCTGATGAGGGT

1501  GAGAATGGCTACGTGACTTACAGTATTGCAAACTTAAATCACGTGCCGTTTGTCATTGAC
      ||||| || |||||||| |||||||| ||||| |||||||| ||||||||||  ||||||
1498  GAGAACGGGTACGTGACATACAGTATCGCAAATTTAAATCATGTGCCGTTTGCGATTGAC

1561  CACTTCACGGGAACCGTGAGTACCTCTGAGAATCTGGACTATGAACTGATGCCTCGCGTG
      || ||||| ||  |||||||||| || || || |||||||| |||||||||||||| ||
1558  CATTTCACTGGTGCCGTGAGTACGTCAGAAAACCTGGACTACGAACTGATGCCTCGGGTT

1621  TACACGCTGAGGATCCGTGCGTCTGACTGGGGCTTGCCATACCGCCGGGAAGTCGAAGTC
      || || |||||||| ||||| || |||||||||||||| |||||||||||||||||||||
1618  TATACTCTGAGGATTCGTGCATCAGACTGGGGCTTGCCGTACCGCCGGGAAGTCGAAGTC
```

```
1681 CTCGCCACGATTACTCTCAATAACCTGAATGACAACACACCCCTGTTTGAGAGGATAAAC
     || || || ||||||||||||||| ||||||||||||||||  |||||||||  |||||
1678 CTTGCTACAATTACTCTCAATAACTTGAATGACAACACACCTTTGTTTGAGAAAATAAAT

1741 TGTGAGGGGACCATTCCCCGAGACCTAGGTGTGGGGGAGCAGATAACCACCGTGTCTGCC
     ||||| ||||| |||||| |||| ||||| ||||| ||||| |||||||| || |||||
1738 TGTGAAGGGACAATTCCCAGAGATCTAGGCGTGGGAGAGCAAATAACCACTGTTTCTGCT

1801 ATTGACGCCGACGAGCTGCAGCTGGTCCGGTACCAGATTGAAGCCGGAAATGAGTTGGAT
     ||||| || || || || ||| |||| | ||| ||||||||||| ||||||||  |||||
1798 ATTGATGCAGATGAACTTCAGTTGGTACAGTATCAGATTGAAGCTGGAAATGAACTGGAT

1861 TTGTTTGGCTTAAACCCCAGCTCCGGTGTGCTGTCCTTAAAGCACTCCCTCACGGATGGC
     || ||| | |||||||||| ||| || ||  |||| |||||||  || || | |||||||
1858 TTCTTTAGTTTAAACCCCAACTCGGGGGTATTGTCATTAAAGCGATCGCTAATGGATGGC

1921 TTGGGCGCAAAGGTTTCCTTTCACAGTTTGAGAATCACAGCGACAGACGGAGAAAATTTT
     || || |||||||| || || |||||| ||||||||||||| ||||| ||||||||||||
1918 TTAGGTGCAAAGGTGTCTTTCCACAGTCTGAGAATCACAGCTACAGATGGAGAAAATTTT

1981 GCCACGCCGTTATACATCAACCTAACGGTGGCTGCCAGTCGCAAGCCGGTCAACTTGCAG
     ||||| || ||||| |||||| |||| ||||||||||||| ||||| ||| |||||||||
1978 GCCACACCATTATATATCAACATAACAGTGGCTGCCAGTCACAAGCTGGTAAACTTGCAG

2041 TGCGAGGAGACCGGTGTTGCCAAAATGTTGGCAGAGAAACTCCTGCAGGCGAATAAGTTA
     || || ||||| ||||||||||||||| |||||||||| ||||||||||| ||||| |||
2038 TGTGAAGAGACTGGTGTTGCCAAAATGCTGGCAGAGAAGCTCCTGCAGGCAAATAAATTA

2101 CACAGTCAGGGGGACGTGGAGGATATTTTCTTTGATTCTTACTCCGTCAATACCCATACT
     ||||  ||||| || ||||||||||||||||| |||||| |||| |||||| | || |
2098 CACAACCAGGGAGAGGTGGAGGATATTTTCTTCGATTCTCACTCTGTCAATGCTCACATA

2161 CCACAGTTCGGGGTTACTCTTCCAACTGGAATTGAGGTAAAGGAGAACCTCCCGGTGGGT
     || |||||  |    |||||||| ||||| ||| |||||||||| ||||  || ||||||
2158 CCGCAGTTTAGAAGCACTCTTCCGACTGGTATTCAGGTAAAGGAAAACCAGCCTGTGGGT

2221 GCCAATATACTCTTCATGAATGCTACTGACCTTGACTCTGGCTTCAATGGAAAGCTCGTC
      ||| | || | ||||||||  | |||||||||||| |||||||||||||||| || |||
2218 TCCAGTGTAATTTTCATGAACTCCACTGACCTTGACACTGGCTTCAATGGAAAACTGGTC

2281 TATGCTATTTCTGGAGGGAATGATGATAGTTGCTTTACCATCGACATGGAAACGGGAGTG
     |||||| |||||||||| ||||| ||||||||||| |  || || |||||||| ||| ||
2278 TATGCTGTTTCTGGAGGAAATGAGGATAGTTGCTTCATGATTGATATGGAAACAGGAATG

2341 CTGAAAGTCCTGTCTCCACTTGACCGTGAAGTAATGGACAAATACACCCTGAATATTACC
     |||||| |  | ||||| ||||||||||||  ||  ||||||||||||||||||||||||
2338 CTGAAAATTTTATCTCCTCTTGACCGTGAAACAACAGACAAATACACCCTGAATATTACC

2401 GTGTACGACCTCGGTATACCCCAGAGGGCTGCCTGGCGCCTTCTGGACGTCACGGTTCTG
     || || ||||| || |||||||||| |||||| ||||| |||||  | |||  |||| |
2398 GTCTATGACCTTGGGATACCCCAGAAGGCTGCGTGGCGTCTTCTACATGTCGTGGTTGTC

2461 GATGCCAATGACAATGCGCCCGAGTTTTTACAGGAGAGCTATTTTGTGGAAGTGAGTGAA
     ||||||||||| ||| | ||||||||||||||||||||||||||||||||||||||||||
2458 GATGCCAATGATAATCCACCCGAGTTTTTACAGGAGAGCTATTTTGTGGAAGTGAGTGAA
```

```
2521 GACAAGGAGGTGAACAGTGAAATCATCCAGGTCGAAGCCACTGATAAAGATCTGGGCCCC
     |||||||||||  | ||||||||||||||||| |||||||| |||||||| ||||| |||
2518 GACAAGGAGGTACATAGTGAAATCATCCAGGTTGAAGCCACAGATAAAGACCTGGGGCCC

2581 AGCGGGCATGTGACCTACGCTATTCTCACAGATACGGAGAAGTTTTCCATCGACAGCATG
     | ||| || ||||| ||| | ||| | ||||| || || |  ||||| || |||||| ||
2578 AACGGACACGTGACGTACTCAATTGTTACAGACACAGACACATTTTCAATTGACAGCGTG

2641 ACCGGTGTGGTGAAAATTATCCAGCCTTTGGATCGTGAAGTGCAACCTGTGCATTACCTA
     || ||||| || || ||    |  ||| ||||||| ||  |||| | || ||| | | ||
2638 ACGGGTGTTGTTAACATCGCACGCCCTCTGGATCGAGAGCTGCAGCATGAGCACTCCTTA

2701 AAGATCGAGGCCAGGGACCAAGCCACAGAGGAACCCCGGTTGTTCTCCACCGTGCTCCTG
     ||||| ||||||||||||||||||| ||| || || | | |||||||||| ||  |  ||
2698 AAGATTGAGGCCAGGGACCAAGCCAGAGAAGAGCCTCAGCTGTTCTCCACTGTCGTTGTG

2761 AAGGTGTCACTAGATGACGTTAATGACAACCCACCTAGGTTCATCCCACCCAATTACTCA
     || || |||||||| || |||||||||||||||||  || || ||||| |||||    
2758 AAAGTATCACTAGAAGATGTTAATGACAACCCACCTACATTTATTCCACCTAATTATCGT

2821 GTGAAAGTTCGAGAAGATCTACCAGAAGGAACCATCATCATGTGGTTAGAAGCCTATGAT
     |||||||| ||||| ||||| |||||||||||| |||||||||||||||||| | |||
2818 GTGAAAGTCCGAGAGGATCTTCCAGAAGGAACCGTCATCATGTGGTTAGAAGCCCACGAT

2881 CCCGATGTGGGTCAGTCAAGTCAGGTGAGATACAGCCTCCTGGATCACGGAGAAGGCCAC
     || ||| | ||||||||  ||||||||||||||||||| ||||| |||||||||||  ||
2878 CCTGATTTAGGTCAGTCTGGTCAGGTGAGATACAGCCTTCTGGACCACGGAGAAGGAAAC

2941 TTTGATGTGGATAAACTCAGCGGCGCAGTTAGAATTGTCCAGCAGCTGGACTTTGAGAAG
     || ||||||||||||||||| || |||||||| || ||||||||| |||||||||||||||
2938 TTCGATGTGGATAAACTCAGTGGAGCAGTTAGGATCGTCCAGCAGTTGGACTTTGAGAAG

3001 AAGCAACTGTATAATCTCACCGTGAGGGCCAAAGACAAAGGGAAGCCCGTGTCCCTGTCT
     |||||| ||||||||||||| ||||||||||||||||| || ||||| || || ||||||
2998 AAGCAAGTGTATAATCTCACTGTGAGGGCCAAAGACAAGGGAAAGCCAGTTTCTCTGTCT

3061 TCCACTTGCTATGTGGAAGTGGAGGTCGTTGACGTGAATGAGAACCTACACACTCCGGTG
     || ||||||||||| ||||| ||||| ||||||| ||||||||||||| ||| | || |||
3058 TCTACTTGCTATGTTGAAGTTGAGGTGGTTGATGTGAATGAGAACCTGCACCCACCCGTG

3121 TTCTCCAGCTTTGTGGAGAAGGGTGTAGTGAAAGAAGACGTCCCTACTGGCTCATCAGTC
     || |||||||||||||| |||||   |||||||||||| |  |||  ||| ||||  || 
3118 TTTTCCAGCTTTGTGGAAAAGGGGACAGTGAAAGAAGATGCACCTGTTGGTTCATTGGTA

3181 ATGACCGTGTCAGCTCACGACGAAGACACAGGCAGAGACGGAGAGATCCGGTATTCCATT
     ||||| ||||| ||||| || || ||| |  | ||||| || |||||||| || ||||||
3178 ATGACGGTGTCGGCTCATGATGAGGACGCCAGAAGAGATGGGGAGATCCGATACTCCATT

3241 AGAGATGGCTCTGGAATTGGTGTCTTCAGGATAGATGAAGAAACAG......
     ||||||||||||||  ||||||| ||||  |||| ||||||| ||||
3238 AGAGATGGCTCTGGCGTTGGTGTTTTCAAAATAGGTGAAGAGACAGTTCATT
```

Table S3. Sequences for qRT-PCR and CHIP primers.

Target genes	Forward (5’-3’)	Reverse (5’-3’)
GAPDH	AACGGGAAGCTTGTCATCAA	TGGACTCCACGACGTACTCA
circFAT1(Div)	GAGGTGGTTGATGTGAATGAGAA	GCAGGAGCAAAGCCAAATGTC
circFAT1(Con)	GTGGAAGTGAGTGAAGACA	CAACGACAGTGGAGAACA
circ0000231	GCAGGAGAAGGCTCTGAAAG	ATGGCTTTATGGCTTGTTGG
circ0002837	GGCCTTTCCTGCTTCTCAT	GCAGTGTGTTATCCCTGCTG
circ0002162	GGGGCAATGCACTAGAAAAG	AATCGCTCTTCACCTGTTGAT
circ0007976	TCCATGTGACCATGAGGAAA	CACCATCATCTGTGAGAACCA
circ0001742	GGGATTTGTTTTGTGGGCTA	CACTGGCCTGAACTGTTGAA
FAT1	ACTACGGATGGGGATTCTGGT	TCTGTAAGGATGACATTCACGGT
SOX2	ATGACCAGCTCGCAGACCTAC	TTGACCACCGAACCCATGGAG
KLF4	GAAATTCGCCCGCTCCGATGA	CTGTGTGTTTGCGGTAGTGCC
c-MET	CGAAAGATAAACCTCTCATAATGA	ATTAAACACTTCCTTCTTTGTGG
BMI1	TGCTTTGGTCGAACTTGGTG	TTTGCAGACTGGGGACAATG
CD24	TGAAGAACATGTGAGAGGTTTGAC	GAAAACTGAATCTCCATTCCACAA
OCT4	TCCCATGCATTCAAACTGAGG	CCTTTGTGTTCCCAATTCCTTCC
ALDH1	GTTCCTGGTTATGGGCCTAC	CCTGGATGCGGCTATACAAC
CXCL9	GTGGTGTTCTTTTCCTCTTGGG	ACAGCGACCCTTTCTCACTAC
CXCL10	GCAAGCCAATTTTGTCCACG	ACATTTCCTTGCTAACTGCTTTCAG
circFat1 (Div)	GAAGACACAGGCAGAGAC	GAGGAGCAGAAGCAGAAG
circFat1 (Conv)	AGGCGAATAAGTTACACAGT	AGAGTCAAGGTCAGTAGCA
Fat1	GCTGTCATCACTGCGATA	GTTCCTTAGTCACCTCTACC
Gapdh	CTCATGACCACAGTCCATGC	CACATTGGGGGTAGGAACAC
Target genes	Position to transcription start site	Sequences (5‘-3’)
CXCL9 CHIP1	Forward (-1002)	AGCAGTGTAGAAGTGTTCCCTGAT
	Reverse (-721)	AATATCCAGAATCTAGAATGAATT
CXCL9 CHIP2	Forward (-731)	TCTGGATATTAGTCCTTTGTCAGG
	Reverse (-461)	TTTCTACTAGTGGTAAGCTCATTC
CXCL9 CHIP3	Forward (-219)	TGTGCCAAAGGCTATCAGTG
	Reverse (-117)	CAGATCCAAGGGAATTTCTGC
CXCL10 CHIP1	Forward (-1111)	TGCAAAGAACACAACCAAGGACCA
	Reverse (-846)	GACACTGTTAAATGCAAAGAAA
CXCL10 CHIP2	Forward (-615)	ACACACCACAAATCAGATACCCAA
	Reverse (-353)	GGTAAACCTAATTTTTTGGTAACAG
CXCL10 CHIP3	Forward (-363)	TAGGTTTACCTATAAAGGATGAA
	Reverse (-92)	ACTCGAAGGTATTATTTATTGTAG
